# Iron Nitride Thin Films: Growth, Structure, and Properties

**DOI:** 10.1021/acs.cgd.1c01528

**Published:** 2022-05-25

**Authors:** Paweł Wojciechowski, Mikołaj Lewandowski

**Affiliations:** † NanoBioMedical Centre, Adam Mickiewicz University, Wszechnicy Piastowskiej 3, 61-614 Poznań, Poland; ‡ Institute of Molecular Physics, Polish Academy of Sciences, M. Smoluchowskiego 17, 60-179 Poznań, Poland

## Abstract

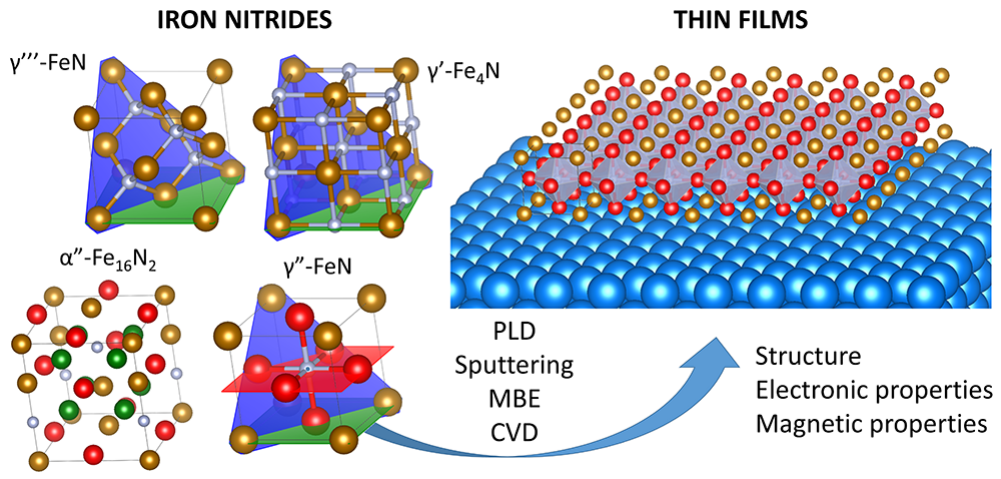

The current state-of-the-art in the growth, structure, and physicochemical properties of iron nitride thin films is presented. First, different iron nitride phases are introduced based on their crystallographic structure and the Fe–N phase diagram. Second, preparation methods for thin iron nitride films are described. Next, the structure, electronic, and magnetic properties of the films are discussed. Finally, potential applications of iron nitride films, as well as the challenges to be faced in the field, are highlighted. This Review constitutes a starting point for anyone who would like to conduct research on these fascinating materials, the scientific and technological potential of which has not been fully explored to date.

## Abstract 2

The review presents the current state-of-the-art in the growth, structure, and physicochemical properties of iron nitride thin films. It constitutes a starting point for anyone who would like to conduct research on these fascinating materials, the scientific and technological potential of which has not been fully explored to date.

## 1 Introduction

Nanometric forms of ionic compounds, such as nanoparticles, nanowires, or thin films of metal oxides, nitrides, and sulfides, are known to exhibit unique structural, physical, and chemical properties not observed for the corresponding bulk materials. These properties depend not only on the size and morphology of the material but also on the crystal phase, varying for different crystallographic orientations and planes. In this respect, thin films grown on single-crystal substrates allow tailoring the properties of a material to a specific phase, crystallographic direction, plane, or even surface termination. Thanks to this, they find applications in various industrial fields, such as mechanical industry, nanoelectronics/spintronics, energy storage/conversion, catalysis, and biomedicine.^1^

Among the ionic compounds, those of iron—one of the most common elements found on our planet, which contributes to approximately 6% of Earth’s crust^2−4^—are of particular interest. Its abundance, low toxicity, and relative ease of use in the production of various tools and constructions make iron one of the most important elements in the history of mankind. In 2021 alone, the annual worldwide production of iron ore reached 1.6 billion tons.^5^

Within the variety of iron compounds, iron nitrides are particularly intriguing, as they exhibit superior mechanical^6−8^ and magnetic^9−11^ properties, as well as corrosion resistance.^12,13^ Even though they have been used in the mechanical industry for over a century,^14,15^ they are still not as thoroughly studied as, for example, iron oxides. This is related to the fact that the naturally occurring iron nitrides are generally rare and mostly found in meteorites, where also several iron nitride-based minerals, such as roaldite—which contains nickel and cobalt additions—may be found.^16^ The discovery of iron nitrides in meteorites suggests that the Earth’s core also consists of these compounds.^17−20^

This article collects and orders the variety of articles published on iron nitride thin films, their growth, structure, physicochemical properties, and potential applications in various industrial fields. It extends the work of Nadzri et al. published in conference proceedings in 2019.^21^ The article concentrates, in most parts, on crystalline structures consisting solely of iron and nitrogen. The focus is on fundamental research, however, with possible applications in mind. For information about iron nitride nanoparticles, please refer to the review article by Bhattacharyya.^22^ Ternary transition metal iron nitrides (i.e., nitrides consisting of Fe, N and another transition metal), on the other hand, are described in the work by Tareen et al.,^23^ while those containing rare-earth metals (being promising permanent magnets) are describes in refs (24 and 25,24), and in the review article by Flores-Livas et al.^26^ Those of these compounds that crystallize in a perovskite structure were additionally described by Niewa.^27^ Moreover, Sun et al. summarized information on the stability of inorganic ternary nitrides,^28^ while Schaaf published an extensive review on metal nitrides seen from the mechanical and engineering point of view (nitriding steel by laser irradiation, etc.).^29^

This Review is divided into seven main sections. In the present one (section 1), a portion of general information regarding iron nitrides is provided, including the historical view, information on the natural occurrence of iron nitrides, and a description of their crystallographic structure. In section 2, several methods used for the preparation of thin iron nitride films are described. The main focus is on physical vapor deposition (PVD) methods; however, chemical vapor deposition (CVD) techniques are also briefly described. Section 3 summarizes information on the structure of differently grown iron nitride thin films based on selected literature reports. Further, the electronic (section 4) and magnetic properties (section 5) of some films are described. Section 6 concentrates on ultrathin (<10 nm-thick) iron nitride films, the structure and properties of which critically depend on the interaction with the substrate. In section 7, several possible applications of iron nitride thin films are mentioned. The summary and outlook, including a critical discussion and highlighting the challenges to be faced in the field, are provided in section 8.

### 1.1 Iron Nitrides: Historical View

The very first experiments on the introduction of nitrogen to iron, with the intention of steel enrichment, were carried out at the beginning of the 20th century.^30^ The initially obtained structures were, however, too brittle for industrial applications. It was only in the early 1920s when the procedure for obtaining “useful” nitrided steel was patented. The two metallurgists who are known for introducing steel nitriding are Adolph Machlet from the American Gas Company in Elizabeth, New Jersey, US,^14^ and Adolph Fry from the Krupp Steel Works in Essen, Germany.^15^ Even though the former was a pioneer in the nitriding process, it was the latter whose works received more attention, making Adolph Fry the “father of nitriding”.^31^

There are several advantages of a nitrided steel over a “classical” carbon-based hardened steel. Both nitrogen and carbon significantly increase the hardness and robustness of steel; however, nitriding can be carried out at much lower temperatures. Standard steel hardening relies on the high-temperature phase transition between α-Fe (ferrite—not to be confused with “ferrite”, i.e., MFe_2_O_4_ mixed oxide) and γ-Fe (austenite) phases, which can be made permanent through quenching. The process increases the hardness; however, it also leads to the appearance of a mechanical stress in the bulk of the material. In the case of nitriding, the temperature of the process is much lower and no quenching is involved. As a result, the mechanical stress in the material is substantially lower. The main disadvantage of nitriding is that the furnaces used for the process are more complex and expensive. Also, not every kind of steel is susceptible to nitrogen-based hardening.^31^

In the second half of the 20th century, other potentially applicable properties of iron nitrides were discovered, which was fueled by the development of sophisticated scientific tools that allowed preparation and physicochemical characterization of different crystalline Fe–N compounds. Notably, one of the iron nitride phases—the α′′-Fe_16_N_2_—was found to exhibit an anomalously large magnetic moment per iron atom.^9^ With the remanence magnetization reaching 2 T (as compared to 1–1.5 T of neodymium magnets), this nitride is the strongest permanent magnet discovered to date.^10^ Unfortunately, despite 50 years of studies, the structural stability of the phase still remains an issue.

### 1.2 Crystallographic Structure of Different Iron Nitride Phases

**Figure 1:**
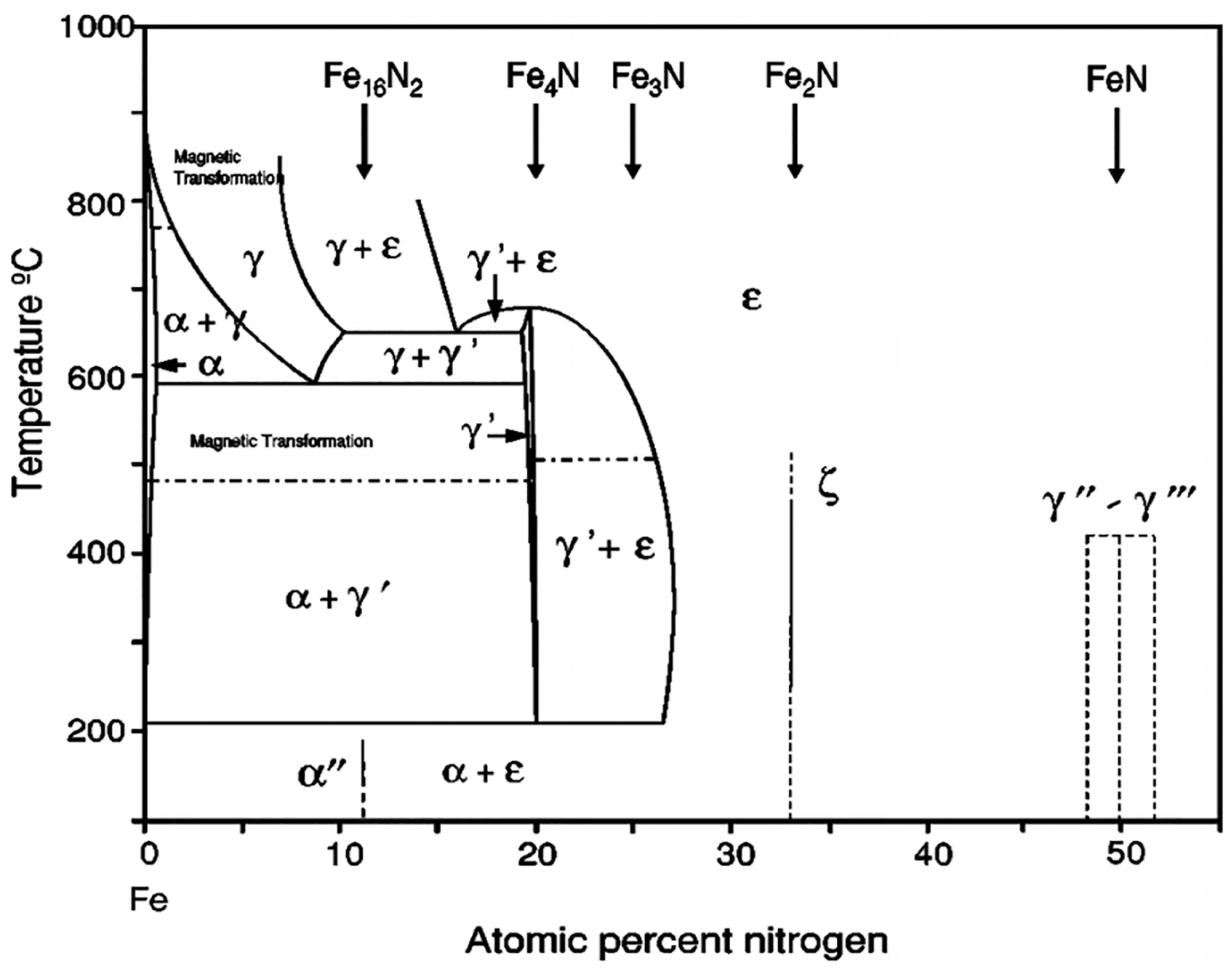
Fe–N phase diagram. Reprinted figure with permission from ref (32). Copyright 2004 by the American Physical Society.

As shown on the phase diagram in Figure 1, several Fe–N phases, differing by the nitrogen content (up to 50%), can be stabilized.^32^ α-Fe and γ-Fe represent different forms of metallic (or slightly nitrogen-doped) iron. As can be seen, some phases may coexist under certain experimental conditions, making the preparation of single-phase samples nontrivial.

#### 1.2.1 γ′′-FeN and γ′′′-FeN

**Figure 2:**
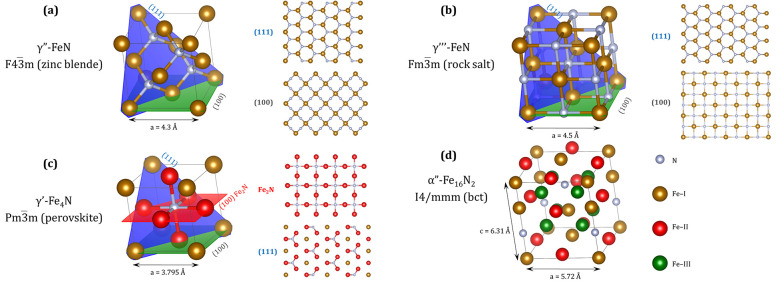
Crystallographic structure of selected Fe–N phases and the top views of their possible low-index surfaces: (a) γ′′-FeN zinc blende structure, (b) γ′′′-FeN rock salt structure, (c) γ′-Fe_4_N perovskite structure, and (d) α′′-Fe_16_N_2_ body-centered tetragonal structure. Gold spheres in (a) and (b) represent Fe atoms, while in (c) and (d) they correspond to Fe-I atoms. Red and green spheres in (c) and (d) represent Fe-II and Fe-III atoms, respectively, while silver spheres correspond to N atoms. The illustration was made using the VESTA software.^43^

With the nitrogen content of around 50%, two phases can be formed: γ′′-FeN and γ′′′-FeN. The former crystallizes in a face-centered cubic (fcc) *F*(4̅)3*m* zinc blende structure (with a lattice parameter *a* = 4.33 Å),^33^ while the latter crystallizes in a *Fm*(3̅)*m* rock salt structure (*a* = 4.57 Å)^34^ (Figure 2a,b). The stability of both phases was confirmed theoretically, with some calculations indicating that the zinc blende structure is more stable,^35−37^ while the other opting in favor of the rock salt structure.^38,39^ Experimentally, Eck et al. observed that, for compounds with stoichiometry FeN_*x*_, the rock salt structure emerges with the nitrogen content *x* = 0.5–0.7, while the zinc blende structure appears for *x* = 0.91.^40^ As far as thin FeN films are concerned, Pandey and Gupta et al. reported a transition from the rock salt γ′′′ to the zinc blende γ′′ structure with increasing film thickness.^41,42^

#### 1.2.2 γ′-Fe_4_N

When the atomic concentration of nitrogen with respect to iron is around 20%, the γ′-Fe_4_N phase forms. This phase crystallizes in a perovskite *Pm*(3̅)*m* structure (lattice parameter *a* = 3.795 Å)^44^ (Figure 2c). Initially, it was believed that the compound represents a solid solution of nitrogen in iron and not a stable iron nitride phase.^45^ However, further X-ray diffraction experiments revealed that nitrogen atoms are placed in well-defined positions and not randomly distributed within the structure.^46,47^ The unit cell hosts two nonequivalent types of iron atoms—Fe-I (second nearest-neighbor of N atom) and Fe-II (nearest-neighbor of N atom). Thus, within the perovskite ABX_3_ structure, the Fe–I atoms occupy the “A” sites, the N atoms the “B” sites, and the Fe-II atoms the “X” sites. When the compound is grown in a form of an ultrathin film (meaning: monolayer in thickness), the stoichiometry is Fe_2_N, as the unit cell represents half of the γ′-Fe_4_N cell.^48^

#### 1.2.3 α′-Fe_8_N and α′′-Fe_16_N_2_

For the nitrogen content of <20%, the α′-Fe_8_N phase (also called iron–nitrogen martensite) preferentially forms. This phase can be considered as a highly distorted α-Fe, with 10% of (0.5, 0.5, 0) and (0, 0, 0.5) sites being randomly occupied by N atoms, which results in a body-centered tetragonal (bct) *I*4/*mmm* structure (with *a* = 2.85 Å and *c* = 3.09 Å; *c*/*a* = 1.08).^49^ Prolonged annealing of the α′-Fe_8_N phase leads to the transformation to α′′-Fe_16_N_2_. In this phase, the nitrogen atoms are located at well-defined positions within the unit cell (the 2a sites—positions (0, 0, 0.33) and (0.5, 0.5, 0.67)). The unit cell is larger (the lattice parameters are *a* = 5.72 Å and *c* = 6.31 Å; *c*/*a* = 1.1)^50^ and consists of eight (2 × 2 × 2) tetragonal cells (Figure 2d). α′′-Fe_16_N_2_ is metastable, susceptible to decomposition and difficult to synthesize in a single-phase form. Usually, it decomposes into α-Fe and γ′-Fe_4_N. Thus, it can be considered as a transition state between α′ and γ′ iron nitrides.

#### 1.2.4 Other Iron Nitride Phases

At the nitrogen content of ∼33%, the ζ-Fe_2_N phase may form, crystallizing in an orthorhombic structure (*Pbcn* space group; lattice parameters *a* = 4.4373 Å, *b* = 5.5413 Å, and *c* = 4.8429 Å).^44^ The unit cell hosts a total number of 12 atoms (8 Fe and 4 N), with iron atoms positioned at the 8d sites *x*, *y*, *z* (ideal parameters: *x* = 0.25, *y* = 0.125, *z* = 0.083) and nitrogen occupying the 4d sites 0, *y*, 0.25 (ideal parameter *y* = 0.375). The first report mentioning this phase dates back to 1928,^51^ when Hägg—studying Fe_*x*_N phases with a high nitrogen content—observed an orthorhombic crystal structure for *x* = 2. The studies of other groups, carried out with the use of X-ray diffraction (XRD),^44^ neutron scattering,^52^ and density functional theory (DFT),^53^ provided information on the atomic positions of atoms inside the unit cell of this compound.

When the nitrogen content is <33%, ε-Fe_*x*_N iron nitrides may possibly form. It is a family of similar compounds with a different nitrogen content (*x* varying between 2 and 3). The occurrence of these phases was first independently reported by Osawa and Iwaizumi,^47^ as well as Hägg^54^ in 1929, while further XRD^55^ and neutron scattering^56^ experiments refined the understanding of their crystal structure. The compounds crystallize in a slightly distorted hexagonal close-packed (hcp) *P*6_3_22 structure with the ABCBA stacking order, where the “A” and “C” layers are filled with Fe atoms, and “B” are occupied by N atoms.^55^ The lattice constants vary depending on the nitrogen content: for *x* = 3 (small nitrogen content) the lattice parameters are *a* = 4.597 Å and *c* = 4.341 Å, while for *x*-2 (high nitrogen content) an expansion of the unit cell up to *a* = 4.787 Å and *c* = 4.418 Å is observed. During the expansion, the structure maintains its crystal symmetry.^55^Table 1 summarizes basic information on the most commonly occurring iron nitrides phases, namely their crystal structure and magnetic properties.

**Table 1:** Basic Information on the Structure and Magnetic Properties of the Most Commonly Occurring Iron Nitride Phases

phase	α-Fe	α′′-Fe_16_N_2_	α′-Fe_8_N	γ′-Fe_4_N	ε-Fe_*x*_N (*x* = 3)	ε-Fe_*x*_N (*x* = 2)	ζ-Fe_2_N	γ′′-FeN	γ′′′-FeN
space group	*Imm*(57)	*I*4/*mmm*^50^	*I*4/*mmm*^49^	*Pmm*(44)	*P*6_3_22^55^	*P*6_3_22^55^	*Pbcn*(44)	*F*43*m*^33^	*Fmm*(34)
lattice constants	*a* = 2.87 Å^57^	*a* = 5.72 Å	*a* = 2.87 Å	*a* = 3.80 Å^44^	*a* = 4.60 Å	*a* = 4.79 Å	*a* = 4.44 Å	*a* = 4.3 Å^33^	*a* = 4.5 Å^34^
		*c* = 6.31 Å^50^	*c* = 3.13 Å^49^		*c* = 4.34 Å^55^	*c* = 4.42 Å^55^	*b* = 5.54 Å^55^		
							*c* = 4.84 Å^44^		
magnetic ordering	ferro-	ferro-	ferro-	ferro-	ferro-	ferro-	ferro-	para-	antiferro-
*T*_C_/*T*_N_	1044 K^57^	813 K^10^	770 K^58^	769 K^57^	558 K^59^	13 K^60^	4 K (bulk)^60^	N/A	100 K^34^
							35 K (thin film)^61^		
μ_Av_/Fe_at_	2.22 μ_B_^57^ [RT]	3.3 μ_B_^10^ [RT]	2.5 μ_B_^58^ [RT]	2.01 μ_B_^62^ [RT]	1.45 μ_B_^59^ [RT]	0.17 μ_B_^63^ [0 K]	0.06 μ_B_ (bulk)^173^	N/A	2.51 μ_B_^35^ [DFT]
							0.028 μ_B_ (thin film)^61^ [0 K]		

**Figure 3:**
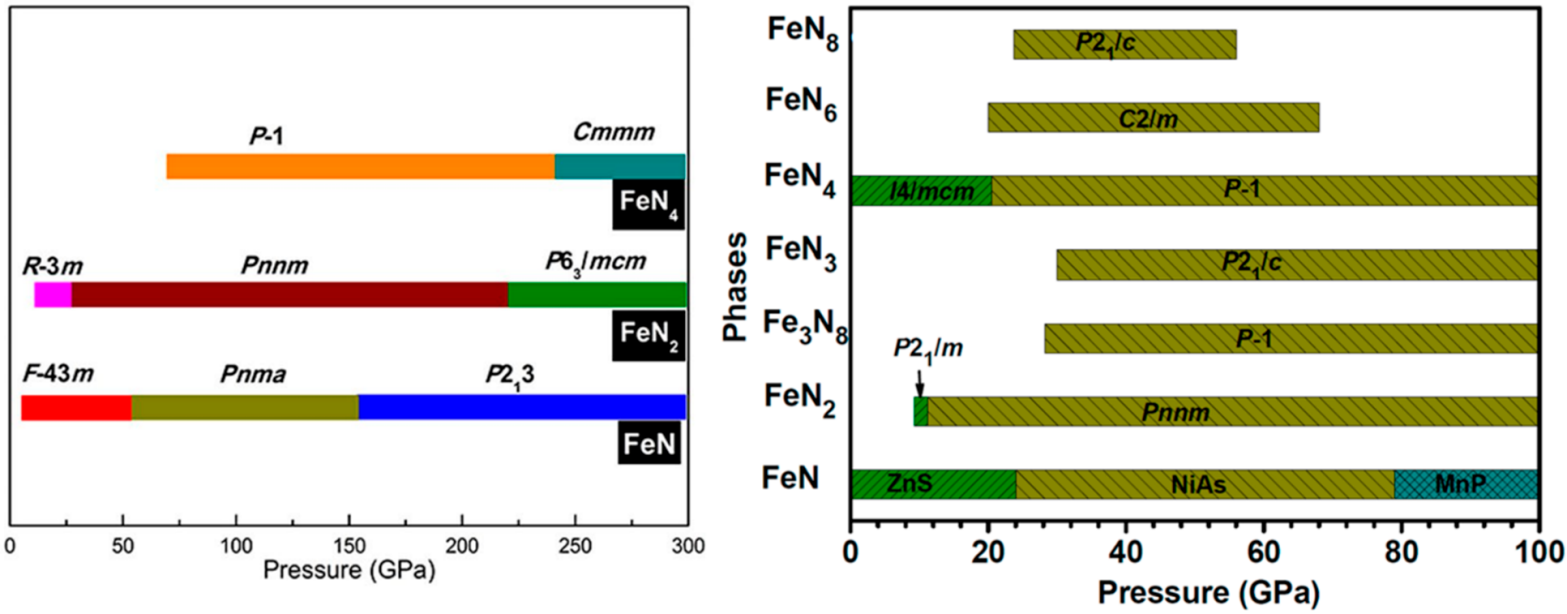
Theoretically calculated high-pressure phase diagrams of iron nitrides. The left image is reprinted with permission from ref (71) under the terms of the Creative Commons CC BY license. Published by Springer Nature. The right image is reprinted with permission from ref (66). Copyright 2018 American Chemical Society.

In addition to the above-mentioned experimentally observed phases, theoretical calculations predict another potentially stable phase—the Fe_3_N_4_.^65^ It should crystallize in a cubic spinel structure (lattice parameter *a* = 7.896 Å) and exhibit weak ferromagnetism (with a magnetic moment per Fe atom equal to 1.09 μB). Also, additional phases with “FeN” stoichiometry were theoretically predicted to be stable, such as wurzite FeN (space group *P*6_3_*mc*),^64^ CsCl-type FeN (*Pm*(3̅)*m*),^35^ and MnP-type FeN (*Pnma*).^66^ However, only the wurzite phase (w-FeN, lattice parameters *a* = 3.77 Å and *c* = 6.05 Å) has been experimentally observed so far.^67^ Moreover, in the high-pressure and high-temperature regime, several additional phases may form: FeN (NiAs-type, *P*6_3_/*mmc*),^68^ FeN_2_ (marcasite *Pnmm*),^69^ β-Fe_7_N_3_ (*P*6_3_*mc*),^17^ and FeN_4_ (*P*(1̅)).^70^ Most of these phases are stable only under high-pressure conditions (from 17 GPa for FeN, up to 135 GPa for FeN_4_). An interesting exception is marcasite FeN_2_ which does not decompose into a nitrogen-poor phase when decompressed from high pressure to ambient pressure but undergoes a phase transition into a *R*(3̅)*m* structure with the same stoichiometry.^69^Figure 3 presents two differently calculated phase diagrams of high-pressure iron nitride phases.^66,71^ It has to be noted that the diagrams differ significantly between each other and are only in partial agreement with the experimental reports (for example, the diagram from ref (71) correctly predicts the formation of *R*(3̅)*m* of FeN_2_, while the pressure at which the transition from the zinc blende FeN to the NiAs-type structure occurs is much more accurately predicted in ref (66)).

## 2 Methods Used for Growing Thin Iron Nitride Films

### 2.1 Pulsed Laser Deposition (PLD)

PLD utilizes highly energetic laser pulses to evaporate material from a metal or metal alloy target. The evaporated materials is then deposited onto a substrate kept at a certain temperature and placed in the vicinity of the evaporation system. For growing metal oxides, sulfides, nitrides, etc., the process is carried out in a reactive atmosphere (such as O_2_, H_2_S, and NH_3_ for metal oxides, sulfides, and nitrides, respectively).

**Figure 4:**
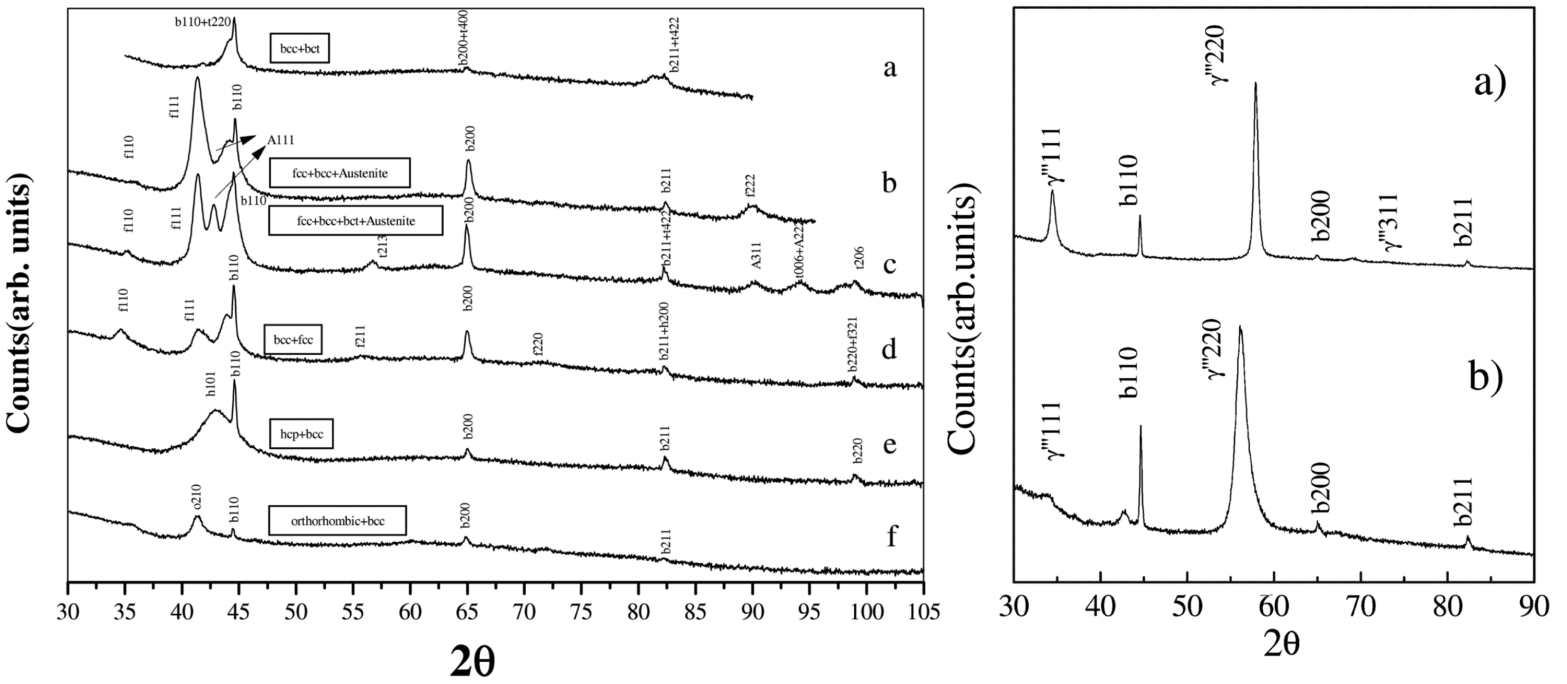
(Left) XRD patterns of iron nitride films grown on glass substrates using PLD of Fe in N_2_ with pressures ranging from 5 × 10^–6^ mbar to 1 × 10^–2^ mbar (a → f); (c) shows the spectrum obtained for the film grown using the same N_2_ pressure as the one in (b) but additionally annealed in nitrogen at 110 °C for 25 h. (Right) XRD patterns of films obtained using GD-PLD (nitrogen plasma) at 200 °C (a) and room temperature (b). Reprinted with permission from ref (72). Copyright 2001 Elsevier.

The PLD technique was successfully applied by various research groups for the growth of thin iron nitride films.^72−75^ The growth usually relies on the introduction of N_2_ into the vacuum system during Fe deposition. The main advantages of this method are the possibility to use low substrate temperatures during film growth (iron nitride formation was reported at 20 °C,^74^ which is much lower compared to other methods, such as molecular beam epitaxy (MBE) (250 °C)^76^ or sputter deposition (300 °C)^77^) and to control the iron nitride phase with the change of nitrogen pressure.^72^Figure 4 (left) shows XRD patterns obtained for thin iron nitride films grown on glass substrates using PLD at different N_2_ pressures ranging from 5 × 10^–6^ mbar to 1 × 10^–2^ mbar. Several different iron nitride phases can be identified based on the observed diffraction peaks: α′′-Fe_16_N_2_ (bct), γ′-Fe_4_N (bcc), γ′′-/γ′′′-FeN (fcc), ε-Fe_*x*_N (hcp), and ζ-Fe_2_N (orthorhombic). What is more, with the use of the so-called glow discharge-assisted PLD (GD-PLD), it was possible to increase the nitrogen content in the samples even more through the formation of a nitrogen plasma. The XRD pattern of a film grown using GD-PLD is presented in Figure 4 (right) and exhibits peaks matching the high-nitrogen-content γ′′′-FeN phase. Unfortunately, all of the patterns also exhibit peaks originating from unreacted iron.

### 2.2 Magnetron Sputtering

Magnetron sputtering deposition (or simply: sputtering) utilizes noble gas ions (typically argon) that hit the target material—which can be a metal, alloy, nonmetal, metal oxide, or other metal/nonmetal compound—ejecting atoms and clusters that are deposited onto a substrate kept at a certain temperature and placed nearby. The energy of the ejected species is low, thanks to which the method can be applied for the deposition of thin films on “soft” substrates (such as polymers). The other advantage of the method is that the evaporated material preserves the stoichiometry of the target, making sputtering a widely used technique for the growth of thin films and their multilayers.^78^ For growing complex compounds, sputtering can be performed, similarly to PLD, in a reactive gas atmosphere (by adding O_2_, N_2_, or other gas to the sputtering noble gas).^79^

There are several reports on the use of sputter deposition for obtaining thin iron nitride films.^77,78,80−88^ The process can be carried out using a dedicated iron nitride target or using pure Fe in a reactive nitrogen-containing atmosphere (in the works included in this Review, only iron deposition in N_2_ was used). Owing to the fact that the method is generally mild to the substrate, the films could be grown on all types of supports: (semi)conducting and insulating, crystalline and amorphous, metal, oxide, polymer, etc. Among the reported cases, films on “classical” silicon wafers,^77^ SrTiO_3_ substrates,^81^ MgO,^82,83,89^ glass,^84^ and polyethylene terephthalate (PETE)^85^ can be found. Also, different types of buffer layers were used to improve the growth of iron nitride films, including noble metals (Ru, Pd, and Pt^88^) and metal nitrides (TiN,^86^ AlN,^87^ Cu_3_N^90^). Most of the obtained films represented the γ′-Fe_4_N and γ′′-FeN phases; however, single works reported the formation of γ′′′-FeN,^91^ ξ-Fe_2_N,^61^ ε-Fe_*x*_N,^80^ and α′′-Fe_16_N_2_.^89^

Recently, a subtype of sputtering, called high-power impulse magnetron sputtering (HiPIMS), was used for iron nitride thin film fabrication.^92,93^ The focus was on the comparison between the structure of Fe_*x*_N films obtained with HiPIMS and the “classical” DC magnetron sputtering. The methods differ with respect to magnetron energy distribution: in DC magnetron sputtering, the sputtering power is constant, while in HiPIMS, pulses with an average power larger by nearly two orders of magnitude are used. This has a significant impact on the properties of the films, both morphological (decreased roughness and thinner interdiffusion region) and magnetic (increased saturation and remanence at similar coercivity).

### 2.3 Molecular Beam Epitaxy (MBE)

**Figure 5:**
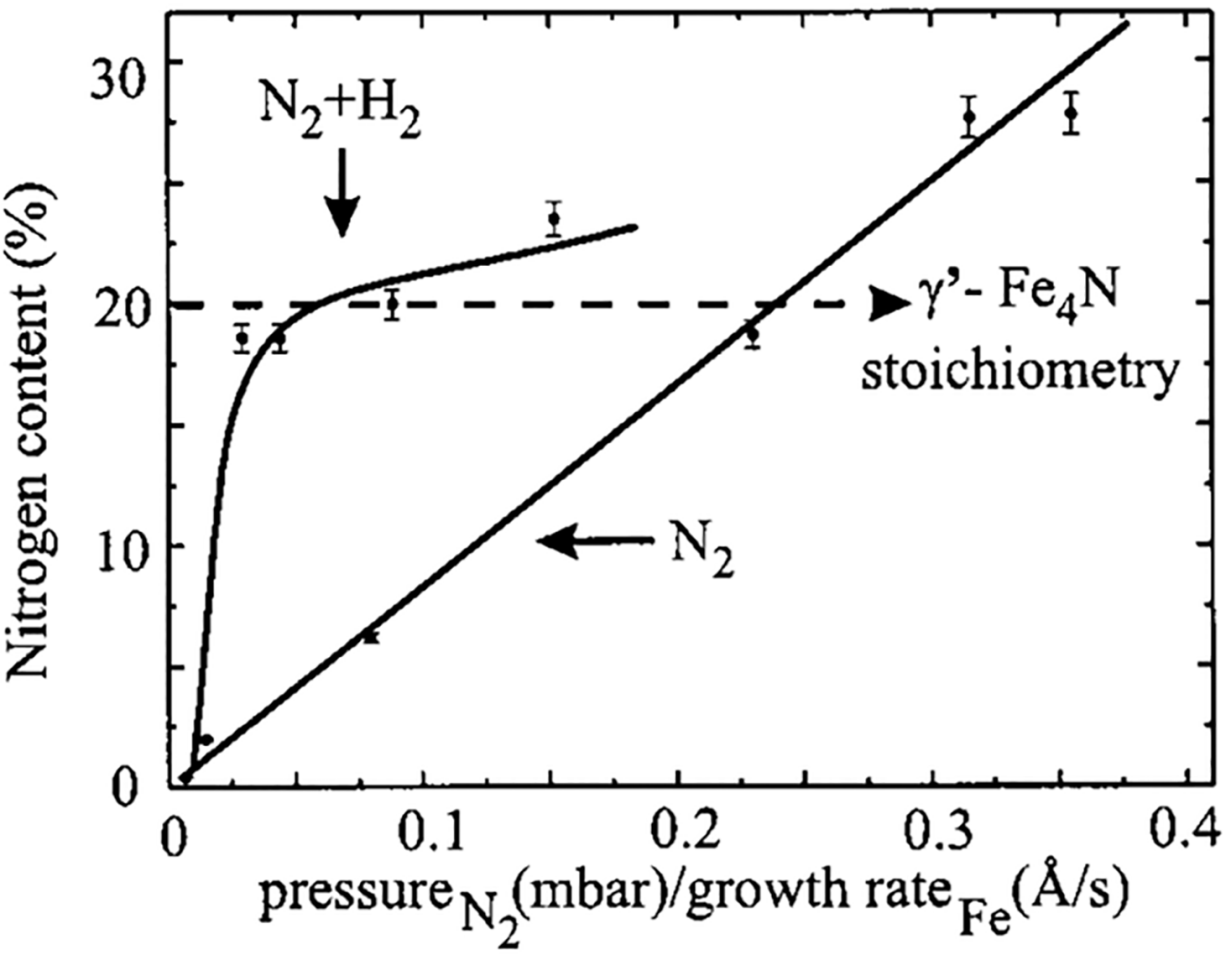
Dependence of the nitrogen content in thin iron nitride films grown by N_2_- and N_2_ + H_2_-plasma-assisted MBE on the nitrogen pressure divided by the iron growth rate. Solid lines are a guide to the eye. Reprinted from ref (95) with permission. Copyright 2001 AIP Publishing.

In MBE, the material is placed in a crucible and heated above the melting point. The evaporating species are being deposited onto a single-crystalline substrate held at a certain temperature and facing the evaporator.^32,94−102^ The deposition speeds are low (e.g., 1 atomic layer per minute), thanks to which the material has time to organize at the surface and adopt the structure of the substrate (resulting in the so-called epitaxial growth). The method was first successfully used for growing thin iron nitride films by Grachev et al., who deposited Fe onto a heated MgO(001) substrate in a nitriding environment.^94^ The authors compared the films grown using different sources of nitrogen: (i) NH_3_ (up to 10^–4^ mbar), (ii) N_2_ + NH_3_ mixture flowing through a hot iron nozzle (leading to the formation of atomic nitrogen), (iii) radiofrequency (RF) nitrogen plasma source used with N_2_, and (iv) RF nitrogen plasma source fed with N_2_ + H_2_. The study proved that the use of a plasma source is the most effective nitriding method among the considered ones. What is more, using a N_2_ + H_2_ mixture has several advantages over pure N_2_. First of all, growing iron nitride films using pure nitrogen was resulting in the appearance of several iron nitride phases at the surface, namely, ε-Fe_*x*_N with different *x* parameters, γ′-Fe_4_N and α-Fe,^94^ while the use of a N_2_ + H_2_ gas mixture led to the formation of single-phase γ′-Fe_4_N films.^96^ The addition of hydrogen is, thus, believed to limit the maximum nitrogen content in the nitride film through the formation of NH_3_ from the excess of nitrogen. Second, the presence of H_2_ was enhancing the growth rate of iron nitride film (Figure 5). Finally, the addition of hydrogen allowed obtaining the same nitrogen content in the film using a much lower pressure.^95^

MBE was also used by Sugita and co-workers for growing thin α′′-Fe_16_N_2_ films on GaAs(001) and In_0.2_Ga_0.8_As(001).^10,103,104^ The epitaxial growth was possible thanks to the small lattice mismatch between the iron nitride and both substrates (*a*_Fe16N2_ = 5.72 Å, *a*_GaAs(001)_ = 5.65 Å, *a*_In0.2Ga0.8As(001)_ = 5.71 Å). Nitriding was performed through the use of a mixture of N_2_ and NH_3_. The obtained iron nitride layers had the thickness ranging from 20 to 100 nm.

There are also a few reports on the MBE growth of iron nitrides on other substrates, such as Cu(001), Cu(111),^76,105^ and MgAl_2_O_4_(001).^106^ Two groups that have relied on this preparation method are particularly prominent: the group of Rodolfo Miranda, which used RF plasma source for nitriding the deposited Fe films (thickness ranging between experiments, from submonolayer to 30 nm^32^), and the group of Fumio Komori, which uses N^+^ ions sputtering for nitriding copper substrates, deposits iron, and anneals to form iron nitrides. Most of the films obtained by these groups represented the γ′-Fe_4_N phase.

To summarize, using MBE, thin films representing the following iron nitride phases were successfully grown: α′′-Fe_16_N_2_ (or, to be more precise, a mixture of α′-Fe_8_N and α′′-Fe_16_N_2_),^102^ γ′-Fe_4_N,^32,95−99^ and γ′′-FeN.^100,101^ Interestingly, on In_0.2_Ga_0.8_As(001), the use of a buffer layer of pure Fe with a thickness of 10–30 nm resulted in a single-phase growth of α′′-Fe_16_N_2_.

### 2.4 Chemical Vapor Deposition (CVD)

Even though the PVD methods dominate when it comes to the growth of iron nitride films, CVD techniques are also utilized for their preparation.^107−116^ The advantage of CVD is that the method does not require high-vacuum chambers (ordered iron nitride films have been grown even in an atmospheric pressure setup^109,111−113^). Funakubo et al. grew iron nitride films using a mixture of ferrocene (Fe(C_5_H_5_)_2_), NH_3_, H_2_, and CO_2_.^109^ In this case, ferrocene constituted the source of iron atoms (the bonds between Fe atoms and cyclopentadiene (Cp) rings are much weaker than those within the hydrocarbon rings; therefore, during thermal decomposition the Fe–Cp bonds break first), while ammonia was the nitriding agent. The addition of hydrogen allowed binding the excess of nitrogen produced in the process, and the presence of carbon dioxide was found to lead to the reduction of carbon content in the fabricated films. The gas mixture was applied to fused silica substrates at different temperatures ranging from 550 to 700 °C. The authors managed to obtain films representing pure γ′-Fe_4_N and ε-Fe_*x*_N phases, as well as mixtures of those.

Besides ferrocene, also iron chloride (FeCl_3_) was successfully used as an Fe source for growing thin iron nitride films.^111−113^ The precursor was mixed with NH_3_, which again acted as the nitrogen source, as well as N_2_ or H_2_ that constituted a carrier gas. The obtained iron nitride phase was found to depend on the substrate type: when glass substrate was used, the ε-Fe_*x*_N phase formed, while the MgO(100) substrate promoted the growth of γ′-Fe_4_N. This is most probably related to the amorphous versus crystalline character of the substrates and the substrate-induced epitaxial growth in the case of MgO.

Iron nitride films can be also grown using atomic layer deposition (ALD)^117^—a variant of CVD that provides uniform surface coverage regardless of the substrate morphology, as well as superior control of film thickness owing to the self-limiting character of the process. The former property constitutes a significant advantage over PVD methods which require a clear line of sight between the source and the surface (“face-to-face” growth), as well as other CVD methods which are not as precise in covering objects with complex surface topography. The self-limiting character of the method, on the other hand, allows obtaining films that suffer less from epitaxial stress, compared to their PVD counterparts. The disadvantages of ALD include the necessity to perform processes at relatively high substrate temperatures—which may be disastrous for some iron nitride phases—as well as sophisticated reaction chambers. The reactants used for growing iron nitride films have been bis(*N*,*N*′-di-*tert*-butylacetamidinato)iron-(II) (Fe(^t^Bu-amd)_2_)) and anhydrous hydrazine, and the substrate was Si(100) covered with a 100 nm-thick amorphous SiO_2_ layer. XRD data recorded for the film grown at 290 °C revealed peaks that match the ε-Fe_3_N phase. X-ray photoelectron spectroscopy (XPS) showed negligible amounts of carbon and oxygen, indicating the high purity of the obtained films. By studying the influence of precursor injection times on the film growth rate, as well as the dependence of the film thickness on the number of cycles, the self-limiting character of the reaction was confirmed. The additionally performed first-principle density functional theory (DFT) calculations revealed that the reaction energy is negative, indicating its spontaneous character. To prove the uniformity of the coverage independently of the substrate morphology, the authors deposited the film onto a substrate covered with 500 nm deep trenches and recorded cross-sectional scanning electron microscopy (SEM) images. The results revealed the uniform thickness of the film at all parts of the surface.

## 3 Structure of Iron Nitride Films

Iron nitride thin films grown using different methods and on different substrates do not only differ by the phase(s) they represent but also the level of crystallinity and the morphology. The thickness of the films reported so far ranges from a single unit cell to hundreds of nanometers. Table 2 summarizes, in chronological order, information on selected iron nitride films, including the phase their represent, thickness, substrate, growth method, main structural features, and the methods used for their characterization. It does not include all the works on the topic (which are >500); however, it provides a good statistical dataset.

In most reported cases, the γ′-Fe_4_N phase was obtained. The films were found to grow in the [001] direction independently of the crystal structure and the orientation of the substrate. The reports on the growth of this phase in different crystallographic directions are extremely rare (see, for example, ref (118)). Single-phase and single-crystal films are only possible for epitaxial growth on single-crystal substrates, such as MgO(001),^94^ Cu(001),^96^ Cu(111),^76^ LaAlO_3_(001),^119^ SrTiO_3_(001),^81^ or Ru(0001) thin film.^88^ The other relatively commonly occurring phases are the γ′′-FeN,^41^ ε-Fe_3-x_N^80^ and α′′-Fe_16_N_2_.^104^ The films representing ζ-Fe_2_N,^120^ α′-Fe_8_N,^106^ γ′′′-FeN,^91^ and w-FeN^67^ constitute very rare cases. Single-phase and single-crystal γ′′-FeN films were obtained on Cu(001),^121^ Fe(001),^101^ and w-GaN(0001).^122^ The growth of single-phase ε-Fe_3−x_N films was achieved on glass,^80^ SiO_2_,^117^ and MgO(111);^80^ however, these films are usually polycrystalline. For growing α′′-Fe_16_N_2_, InGaAs(001),^10^ GaAs(001),^104^ Fe/MgO(001),^102^ and Fe/MgAl_2_O_4_(001)^106^ constitute the best substrates. It has to be mentioned that in some cases the film can be transformed into the α′-Fe_8_N phase through vacuum annealing. Generally, it is evident that noncrystalline supports, such as glass,^72^ fused silica,^109^ or PETE,^85^ promote multiphase and polycrystalline growth of iron nitrides. As far as the growth method is concerned, most of the listed iron nitride films were grown using magnetron sputtering and MBE, while PLD,^72^ CVD,^115^ and nitriding of single-crystal iron substrates^101^ were used in few works only. The most common way to obtain single-phase films is to use Fe deposition in the presence of nitrogen plasma and with hydrogen in the reactor (that allows binding the excess of nitrogen). The methods most commonly used for the determination of films structure are XRD, SEM, TEM, AES, XPS, and AFM, providing information on the crystalline phase (XRD, TEM), surface topography (SEM, AFM), and chemical structure (AES, XPS).

It is also important to mention that the structure of the film may evolve with thickness. Recently, Pandey and Gupta et al. studied the growth of thin FeN films using the X-ray absorption near-edge structure (XANES) technique, XRD, XRR, and nuclear resonant scattering (NRS).^41,42^ The films were grown on Si substrates (crystallographic orientation not specified) and SiO_2_ (amorphous). The authors observed changes in the N K-edge fine structure with increasing film thickness from 5 to 10 nm. These changes were attributed to the phase transition between rock salt and zinc blende FeN structures (the critical thickness was found to depend on the sample temperature). Studies were also carried out for films with a thickness ranging from 2 to 2500 Å that were grown on a quartz substrate. The thinnest film (2 Å) was characterized by XANES spectra that dramatically differed from those obtained for thicker samples and resembling that of molecular nitrogen. Thicker layers, between 4 and 150 Å, hosted a mixture of molecular nitrogen and zinc blende iron nitride. Above 1000 Å, the films were resembling the structure of bulk iron nitride.

## 4 Electronic Properties

In general, iron nitrides are electrically conducting; however, they differ with respect to their detailed electronic structure. Extensive work has been carried out to calculate the electronic properties of various iron nitride phases.^65,137−146^ Here, the basic mechanisms responsible for the changes in the electronic structure of different Fe−N compounds are presented together with experimental results published in the literature for thin film systems.

**Table 2:** Overview of the Literature Data on the Structure of Thin Iron Nitride Films Grown Using Different Methods

ref	iron nitride phase	film thickness [Å]	substrate	growth method	structure	characterization methods	year
(9)	α′′-Fe_16_N_2_	500	glass	MBE of Fe in N_2_	polycrystalline, multiphase (no detailed description)	magnetometry, RHEED	1972
	γ′-Fe_4_N						
(107)	ε-Fe_3–2_N	50000	glass	CVD using Fe(CO)_5_, NH_3_, H_2_, and Ar	amorphous at 50 °C; columnar structure at 200 °C	SEM, XRD, magnetometry	1986
(108)	γ′-Fe_4_N^a^	300–600	glass	CVD using HFe_4_(CO)_12_N	amorphous (no detailed description)	XRD, XPS, AES, CEMS	1990
(109)	γ′-Fe_4_N	No data	fused silica	CVD using Fe(C_5_H_5_)_2_, NH_3_, H_2_, and CO_2_	polycrystalline, grain structure	XRD, VSM, SEM	1990
	ε-Fe_3–2_N						
(10)	α′′-Fe_16_N_2_	500, 1000 (100–300 with Fe buffer layer)	InGaAs(001)	MBE of Fe in N_2_ + NH_3_ (pure N_2_ for Fe buffer layer)	epitaxial, single-crystal, single-phase	VSM, TEM, RHEED, XRD	1991
(103)	α′′-Fe_16_N_2_	200–900	InGaAs(001)	MBE of Fe in N_2_ + NH_3_	single-crystal, single-phase	XPS, AES, TEM, XRD, RHEED, VSM, CEMS	1994
	α′-Fe_8_N						
(123)	α′-Fe_8_N	200–850	Ag/Fe(3 nm)/MgO(001)^b^	DC magnetron sputtering of Fe in N_2_ + Ar (α′), annealing in N_2_ (α′′)	epitaxial, multiphase	XRD, VSM, CEMS (parameters given, but no spectra included)	1994
	α′′-Fe_16_N_2_						
(104)	α′′-Fe_16_N_2_	300–900	GaAs(001)	MBE of Fe in N_2_ + NH_3_	epitaxial, single-crystal, single-phase	RBS, FMR, SQUID, VSM, DC four-point probe electric measurements	1996
(120)	ζ-Fe_2_N	5–300^c^	Si(001)	RF magnetron sputtering of Fe in N_2_ + Ar	polycrystalline, single-phase	XRD, SAXS, TEM, SQUID	1996
(110)	ε-Fe_3_N	∼10000	polycrystalline Ti	CVD using iron acetylacetate, NH_3_, and N_2_	polycrystalline, grain structure	XRD, SEM, EDX	1998
(111)	ε-Fe_3_N	900–5000	glass	CVD using FeCl_3_, NH_3_, and N_2_	smooth, single-phase (no detailed description)	XRD, SEM, VSM	1999
(112)	ε-Fe_3_N	10000–50000	glass	CVD using FeCl_3_, NH_3_, and N_2_	single-phase (no detailed description)	XRD, SEM, VSM	2000
(72)	α′′-Fe_16_N_2_	no data	glass	PLD of Fe in N_2_, GD-PLD of Fe in a nitrogen plasma	multiphase (no detailed description)	XRD, CEMS	2001
	γ′-Fe_4_N						
	γ′′′-FeN						
	ε-Fe_3_N						
	ζ-Fe_2_N						
(84)	FeN^d^	1310	glass	sputtering of Fe in N_2_ + Ar	amorphous, grain-structure (typical grain size ∼30 nm), RMS roughness <1 nm	CEMS, XRD (no pattern included), AFM, XPS, XRR	2001
(113)	γ′-Fe_4_N	No data	MgO(001)	CVD using FeCl_3_, NH_3_, and N_2_	epitaxial, single-phase, RMS roughness 0.5 nm	XRD, SEM, AFM, VSM, TEM, light reflectivity	2001
(94)	γ′-Fe_4_N	300–1000	MgO(001)	MBE of Fe in NH_3_, NH_3_ passed through a hot nozzle and RF N_2_ or N_2_ + H_2_ plasma	epitaxial, single-crystal, single-phase	XRD, CEMS, ERD, RBS	2002
(74)	γ′-Fe_4_N	1140–2350	SiO_2_(300 nm)/Si(001)	PLD of Fe in N_2_	multiphase (no detailed description)	XRD, VSM, AFM (no image included), XPS (no spectra included)	2003
	ε-Fe_3_N						
(96)	γ′-Fe_4_N	2–10 (up to 5 MLs)	Cu(100)	MBE of Fe in RF N_2_ + H_2_ plasma	single-phase, single-crystal; <1 ML: embedded islands; 1 ML: islands, 2.2 Å high; >1 ML: films with 0.5 and 1.9 Å steps and *a*_(100)_ = 3.8 Å	LEED, AES, STM, XRD, CEMS	2003
(102)	α′-Fe_8_N	65, 160, 330, 420	MgO(001), Fe(42 nm)/MgO(001)	MBE of Fe in N_2_, postnitriding in N_2_ (α′′)	no detailed description	CEMS	2003
	α′′-Fe_16_N_2_						
	γ′-Fe_4_N						
	γ′′-FeN						
	γ′′′-FeN						
	ε-Fe_*x*_N						
(32)	γ′-Fe_4_N	<1 ML (2 Å) up to 270 MLs (∼1000 Å)	Cu(100)	MBE of Fe in RF N_2_ plasma	<1 ML: embedded islands; >1 ML: terraces, flat surface	STM, AES, XRD, LEED, CEMS	2004
(97)	γ′-Fe_4_N	<5 (up to 2.3 MLs)	Cu(100)	MBE of Fe in RF N_2_ + H_2_ plasma	epitaxial, square islands with lateral sizes of ∼10 nm	STM, LEED, AES	2004
(61)	ζ-Fe_2_N	1200	SiO_2_/Si(001)	DC magnetron sputtering of Fe in N_2_ + Ar	polycrystalline, grain structure (20 nm on average) + iron oxide	XRD, TEM, XPS (no spectra included), SQUID	2004
(124)	γ′-Fe_4_N	400	Cu(100)	MBE of Fe in RF N_2_ plasma	epitaxial, single-phase, single-crystal, atomically flat surface	STM, LEIS, DFT	2005
(98)	γ′-Fe_4_N	18	Cu(100)	MBE of Fe in RF N_2_ plasma	reconstructed *p4gm*(2 × 2) and unreconstructed (bulk-like) surface regions	STM, LEED, XRD, XPS, UPS, AES, DFT	2007
(99)	γ′-Fe_4_N	up to 500	Cu(001)	MBE of Fe in RF N_2_ + H_2_ plasma	smooth surface regardless of the film thickness	STM, AES, LEED, MOKE	2007
(100)	γ′′-FeN	9	MgO(001)^e^, Cu(001)	MBE of Fe in RF N_2_ plasma	single-phase (no detailed description)	LEED, XPS, UPS, AES, XRD, DFT	2008
(125)	γ′-Fe_4_N	up to 500	Cu(100)^f^	MBE of Fe in RF N_2_ plasma	epitaxial, single-phase, single-crystal, atomically flat surface	STM, LEED, MOKE	2008
(78)	γ′-Fe_4_N	100–500	Si(001)^g^	DC magnetron sputtering of Fe in N_2_ + Ar	grain structure with sizes up to 20 nm (no detailed description)	XRD, DC four-point probe electric measurements, SIMS	2009
(114)	γ′-Fe_4_N	3000	SiO_2_(100 nm)/Si(001)	CVD using FeCl_2_, N_2_, H_2_, and Ar	multiphase, grain structure (500 nm for γ′, 350 nm for ε and 100 nm for ζ)	XRD, SEM, SQUID	2009
	ε-Fe_3_N						
	ζ-Fe_2_N						
(121)	γ′-Fe_4_N	32	Cu(100)	MBE of Fe in a flux of atomic nitrogen	no detailed description	XPS, MOKE, LEED, XRD (no pattern included)	2009
	γ′′-FeN^h^						
(126)	ε-Fe_3_N	300–1500	glass	DC magnetron sputtering of Fe in N_2_ + Ar	polycrystalline, grains 5–50 nm, RMS roughness 8–20 nm (both values increasing with film thickness)	XRD, TEM, SEM, AFM, SQUID	2009
	ζ-Fe_2_N						
(77)	γ′-Fe_4_N	40	Si(001)^i^	DC reactive magnetron sputtering of Fe in N_2_ + Ar	no detailed description	DC four-point probe electric measurements	2010
(101)	γ′′-FeN	30	Fe(001)	exposing a Fe(001) substrate to RF N_2_ plasma	single-phase (no detailed description)	LEED, XPS, UPS, XRD, DFT	2010
(73)	γ′-Fe_4_N	2000	Fe(20 nm)/Si(001)	PLD of Fe in N_2_	columnar microstructure with ∼110 nm grains, RMS roughness ∼10 nm	XRD, TEM, SQUID, MOKE	2011
(75)	γ′′-FeN	no data	Al (crystallographic orientation not provided)	PLD of Fe in N_2_	no detailed description	Mössbauer spectroscopy, XRD (phase composition, no pattern included)	2011
	γ′′′-FeN						
(119)	γ′-Fe_4_N	80–300	LaAlO_3_(001), SrTiO_3_(001), MgO(001)^j^	MBE of Fe in RF N_2_ plasma	single-phase, smooth surface with RMS roughness ∼0.25 nm; epitaxial growth only for LaAlO_3_ and SrTiO_3_	RHEED, AFM, TEM, STEM, EELS	2011
(81)	γ′-Fe_4_N	100	SrTiO_3_(001)^k^	MBE of Fe in RF N_2_ plasma	epitaxial, single-phase (no detailed description)	XRD, RHEED, DFT, AES, HX-PES, SRPES	2012
(82)	γ′-Fe_4_N	no data	MgO(001)	DC magnetron sputtering of Fe in N_2_	single-phase, nanosized grain structure, which transforms at 450 °C into a continuous and smooth one with RMS roughness <0.7 nm	XRD, AFM, SEM, VSM, MOMM, SQUID	2012
(127)	γ′′′-FeN	no data	Al (crystallographic orientation not provided)	PLD of Fe in N_2_	single-phase (no detailed description)	XRD, CEMS, DFT	2012
(128)	α′′-Fe_16_N_2_	150^l^	Fe(5 nm)/GaAs(001)	sputtering of Fe in N_2_ + Ar, annealing in N_2_	most probably multiphase (besides α′′, there is a possibility for the presence of α′; no detailed description)	XRD, XRR, VSM	2013
(83)	α′′-Fe_16_N_2_	80–240	MgO(001)^m^	MBE of Fe in N_2_ + H_2_	polycrystalline (no detailed description)	RBS, CEMS	2014
	γ′′-FeN						
	ε-Fe_3_N						
(115)	γ′-Fe_4_N^n^	1000–10000	Si(001)^o^	CVD using Fe[N(*t*-Bu)_2_]_2_ and NH_3_	as deposited: amorphous; after annealing: columnar microstructure (no detailed description)	TEM, XPS, TOF-SIMS	2014
(87)	γ′-Fe_4_N	1200	AlN(25 nm)/glass	DC magnetron sputtering of Fe in N_2_ + Ar	granular structure with 30 nm grains, RMS roughness 3 nm	XRR, PPMS, AFM, MFM, DFT	2015
	γ′′-FeN						
	ε-Fe_3_N						
(92)	γ′-Fe_4_N	800–1000	glass	DC magnetron sputtering of Fe in N_2_ + Ar; HiPIMS in N_2_ + Ar	polycrystalline with crystallite sizes 10–50 nm, single-phase possible	XRD, AFM, PNR, VSM, XAS, SIMS	2015
	ε-Fe_3_N						
(11)	γ′-Fe_4_N	7.6–30.4^p^	MgO(001)	DC magnetron sputtering of Fe in Ar and N_2_ + Ar	epitaxial, single-phase	XRD, VSM	2016
(48)	γ′-Fe_4_N	<2 (1 ML)	Cu(001)	N^+^ sputtering, MBE of Fe, annealing	well-ordered, single-phase	STM, STS, DFT	2016
(129)	α′′-Fe_16_N_2_	5000	Fe(110) foil on Si(111)^q^	N^+^ implantation (100 keV at room temperature), annealing	polycrystalline, multiphase (γ′ and FeSi present), granular structure with average grain size 25–30 nm; darker regions ∼20 nm in size, 140–200 nm apart (probably nitrogen rich regions)	XRD, TEM, VSM	2016
(85)	α′′-Fe_16_N_2_	700–1800	PETE	DC reactive magnetron sputtering of Fe in N_2_ + Ar	columnar structure with grains 4–15 nm in diameter, RMS roughness 4.7–11 nm	XRD, AFM, VNA, VSM, UV–vis, EIS, TEM	2017
	γ′-Fe_4_N						
	ε-Fe_3_N						
	ζ-Fe_2_N						
(88)	γ′-Fe_4_N	100, 200, 400	Ru(0001) thin film^r^	DC magnetron sputtering of Fe in N_2_ + Ar	the RMS roughness for a 10 nm layer was 0.15 nm (no additional information)	XRD, XPS, VSM, AFM, PCAR	2017
(106)	α′-Fe_8_N	530–790	Fe(3 or 10 nm)/MgO(001), Fe(3 or 10 nm)/MgAl_2_O_4_(001)^s^	MBE of Fe in RF N_2_ plasma, annealing in N_2_ (for α′′)	epitaxial, single-crystal, multi- or single-phase (only for α′)	RHEED, XRD, VSM	2017
	α′′-Fe_16_N_2_						
(130)	γ′-Fe_4_N	<2 (1 ML)	Cu(001)	N^+^ sputtering, MBE of Fe, annealing	epitaxial, atomically flat surface, multiphase (Fe_2_N and a hexagonal phase assigned to FeN)	STM, STS	2017
	γ′′-FeN^t^						
(131)	γ′-Fe_4_N	2–6 (1–3 MLs)	Cu(001)	N^+^ sputtering, MBE of Fe, annealing	epitaxial, atomically flat surface, single-phase	STM, STS, XAS, XMCD, DFT	2017
(76)	γ′-Fe_4_N	<2 (1 ML)	Cu(111)	N^+^ sputtering, MBE of Fe, annealing	epitaxial, atomically flat surface, single-phase	STM, XPS, LEED	2018
(132)	γ′-Fe_4_N^u^	1800	Si(111), MgO(5 nm)/Si(111)	DC magnetron sputtering of Fe in N_2_	single-phase (no detailed description)	XRD, XPS, VSM	2018
(80)	ε-Fe_3-*x*_N (0.07 < *x* < 0.87)	1300	glass, MgO(111)	DC magnetron sputtering of Fe in N_2_ + Ar	epitaxial, single-phase, polycrystalline	XRR, XRD, FESEM, TEM, PPMS, DC four-point probe electric measurements	2019
(105)	γ′-Fe_4_N	<2 (1 ML)	Cu(111)	N^+^ sputtering, MBE of Fe, annealing	epitaxial, atomically flat, single-phase, reconstructed *p4gm*(2 × 2)	STM, STS, LEED, XAS, XMCD	2019
(117)	ε-Fe_3_N	400	SiO_2_(100 nm)/Si	ALD using Fe(^t^Bu-amd)_2_ and N_2_H_4_	at lower temperature (up to 265 °C): amorphous, RMS roughness <3 nm; at higher temperature (290 °C): polycrystalline, single-phase, grain structure, RMS roughness 7–30 nm	SEM, XPS, DFT, AFM (surface roughness, no images included), GIXRD (discussed, but no pattern included)	2019
(133)	γ′′-FeN	6300	glass, polycrystalline Cu foil	RF magnetron sputtering of Fe in N_2_ + Ar	single-phase, polycrystalline, grain structure	XRD, SEM, EDS, TEM, XPS, cyclic voltammetry	2019
(41)	γ′′-FeN	17–500	Si, SiO_2_/Si, sapphire^v^	magnetron sputtering of Fe in N_2_	single-phase, roughness 1–2 nm	XRD, XANES, CEMS, XRR, NRS	2019
(93)	γ′-Fe_4_N	500	LaAlO_3_(100)	MBE of Fe in RF N_2_ plasma; DC magnetron sputtering of Fe in N_2_ + Ar; HiPIMS of Fe in N_2_ + Ar	epitaxial, single-phase; the microstructure (roughness, grain distribution, interface quality etc.) depended on the growth method	XRD, RHEED, VSM, XRR, SIMS, PNR, XAS, XMCD, MOKE	2019
(91)	γ′-Fe_4_N	1300	MgO(111)	DC magnetron sputtering in N_2_ + Ar	depends on the growth method, epitaxial and single-phase possible	XRD, XRR (only mentioned, no pattern included), TEM, XPS, PPMS	2019
	γ′′-FeN						
	γ′′′-FeN						
	ε-Fe_3_N						
(67)	γ′′-FeN	200–2000	Si(100)	reactive magnetron sputtering of Fe in N_2_ + Ar	polycrystalline, multiphase, grain size 2.8–9.2 nm (depending on the film thickness)	GIXRD, CEMS, VSM, NRP	2020
	γ′′′-FeN						
	w-FeN						
(134)	γ′-Fe_4_N	<2 (1 ML)	Cu(001)	N^+^ sputtering, MBE of Fe, annealing	epitaxial, atomically flat surface, multiphase (Fe_2_N and a hexagonal phase assigned to FeN)	STM	2020
	γ′′-FeN						
(135)	γ′-Fe_4_N	600	Si(100), amorphous SiO_2_^w^	DC magnetron sputtering of Fe in N_2_ + Ar	polycrystalline, single-phase, roughness 5.4–19 nm (depending on buffer layer)	XRD, VSM, XRR, PNR, SIMS, AFM	2020
(122)	γ′′-FeN	5–1000	w-GaN(0001)	DC magnetron sputtering of Fe in N_2_	epitaxial, single-phase	XRD (only for 1000 Å film), RHEED	2021
(118)	γ′-Fe_4_N	300	MgO(100), MgO(111)	DC magnetron sputtering of Fe in N_2_ + Ar	epitaxial, single-phase, roughness 5–7.5 nm (depending on the substrate)	XRD, MOKE, SIMS, XRR	2021
(42)	γ′′-FeN	2–2500	quartz (crystallographic orientation not provided)	DC magnetron sputtering of Fe in N_2_	no detailed description	XANES	2021
(136)	γ′′-FeN	2430–3140	amorphous SiO_2_	DC magnetron sputtering of Fe in N_2_	polycrystalline, single-phase iron nitride or Ag-doped FeN, grain size 8–31 nm(decreasing with increasing Ag content)	XRD, SEM, CEMS, SIMS, PNR	2022

a It is possible (but not fully confirmed) that the ε-Fe_3_N phase was also present in the studied sample.

b The thickness of the Ag layer was not reported.

c The article describes the preparation and characterization of (Fe/ζ-Fe_2_N) multilayers on a silicon substrate; the thickness of the iron layer was constant, while the thickness range of iron nitride is given in the table.

d The authors were not able to determine whether the film represent the γ′′ or the γ′′′ phase.

e The authors used Cu(100) substrate for measurements in UHV and MgO(001)/Fe_4_N/Cu/FeN/Cu for the studies carried out under ambient conditions.

f The iron nitride was covered with a 3 nm-thick Cu capping layer.

g The iron nitride was a part of a multilayer magnetic tunnel junction: Si(001)/Cu/Fe_4_N/Cu/Fe_4_N/Mg/MgO/Co_42_Fe_38_B_20_/Ru/Fe/Mn_78_Ir_22_/Ru.

h The γ′ phase was used as a reference; the main focus of the article was on the thermal transformation from the γ′′ phase to the γ′.

i The iron nitride was a part of a multilayer magnetic tunneling junction: Si(001)/[buffer layer]/Fe_4_N/Co_40_Fe_40_B_20_/Ru/Fe/Mn_75_Ir_25_ /[capping layer].

j Some samples were covered with a 3 nm-thick Al capping layer.

k On top of the nitrided iron film, a 1 nm-thick CaF_2_ capping layer was deposited.

l The authors presented results obtained for two samples: Fe–N/Fe/MgO and [Fe–N/Fe]_3_/MgO.

m The samples were covered with a 5 nm-thick Cu capping layer.

n The Authors were unable to obtain confirmation regarding the crystallographic phase, however, chemical composition matched the γ′ phase.

o The iron nitride films were covered with HfB_2_ and Pt capping layers.

p The authors prepared several [Fe/Fe_4_N] multilayer samples with varying iron nitride film thickness.

q An iron foil with a thickness of 500 nm (for preparation and characterization, it was placed on a Si(111) substrate).

r Full structure: γ′-Fe_4_N/Ru(0001)/Ta/SiO_2_/Si.

s The samples were covered with a thin (3–4 nm) capping layer of Al or Ti.

t An additional hexagonal structure was observed at the step edges. The authors tentatively assigned it to the γ′′-FeN phase; however, the definite explanation appeared in their next article (ref (134)).

u The aim of the authors was to study the γ′-Fe_4_N phase; however, at certain growth conditions the presence of the α, γ, ε, and ζ phases was also observed.

v Both the crystallographic orientation of the silicon substrate and the oxide thickness were not specified.

w As the substrates, the naturally oxidized silicon or silicon with a Cu, Ag, or CrN buffer layer (50 nm in thickness) was used. The experimental section mentions all the substrates listed; however, the results are presented only for naturally oxidized silicon.

**Figure 6:**
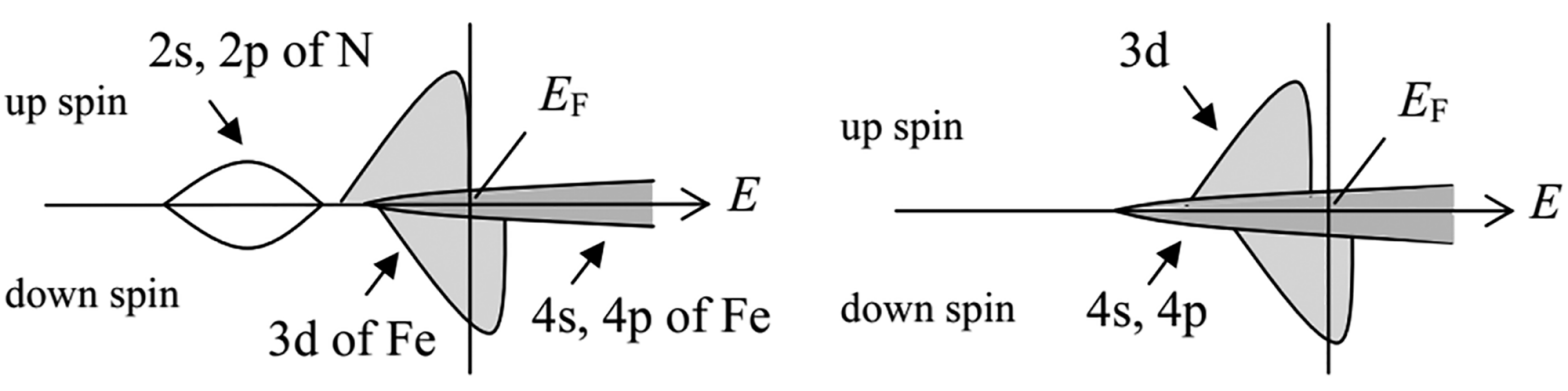
Schematic illustration of partial DOS of Fe_4_N (left) and fcc Fe (right). Reprinted with permission from ref (142). Copyright 2006 John Wiley & Sons.

Coey and Smith studied the influence of the addition of nitrogen to iron on the electronic properties of the system.^147^ The authors found that the nitrogen sp orbitals hybridize with the 3d states of iron, reducing the difference between occupancy of 3d↑ and 3d↓. Interstitial atoms also expand the lattice, reducing the 3d–3d overlap and the bandwidth. Additionally, the appearance of nitrogen 2s and 2p orbitals shifts the 4s and 4p orbitals in energy. The latter might seem negligible due to the low density of states (DOS) of Fe 4s and 4p orbitals compared to d orbitals; however, it leads to large spin polarization at the Fermi level, which is close to −1.0 for the Fe_4_N iron nitride.^142^ A schematic illustration of changes in the electronic structure of Fe induced by the introduction of N is presented in Figure 6.

**Figure 7:**
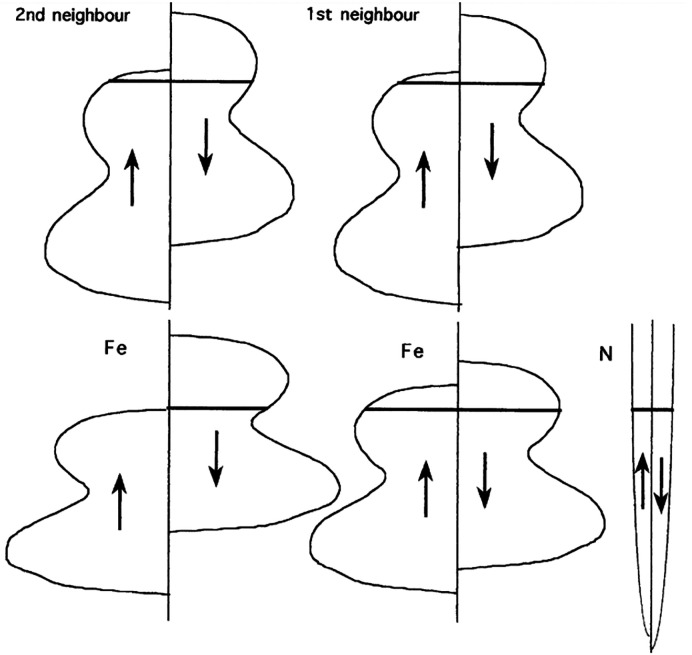
Schematic illustration of changes in the electronic structure of first- and second-neighbor iron atoms in bcc Fe induced by the introduction of a N atom. Reprinted with permission from ref (147). Copyright 1999 Elsevier.

Hybridization also affects the magnetic moments of iron atoms. Overlapping of nitrogen sp states with the nearest-neighbor iron reduces the spin splitting, lowering the potential of 3d↓ electrons. This results in a charge transfer from more distant iron atoms, depleting the 3d↓ band and increasing the magnetic moment. The process is illustrated in Figure 7. It must be noted that even though the magnetic moment of specific iron site may reach 2.7 μB the theory does not allow the average iron moment to be higher than that value.^147^ However, as experiments show, this is not always the case, especially among the nitrogen-poor iron nitride phases.^104^

**Figure 8:**
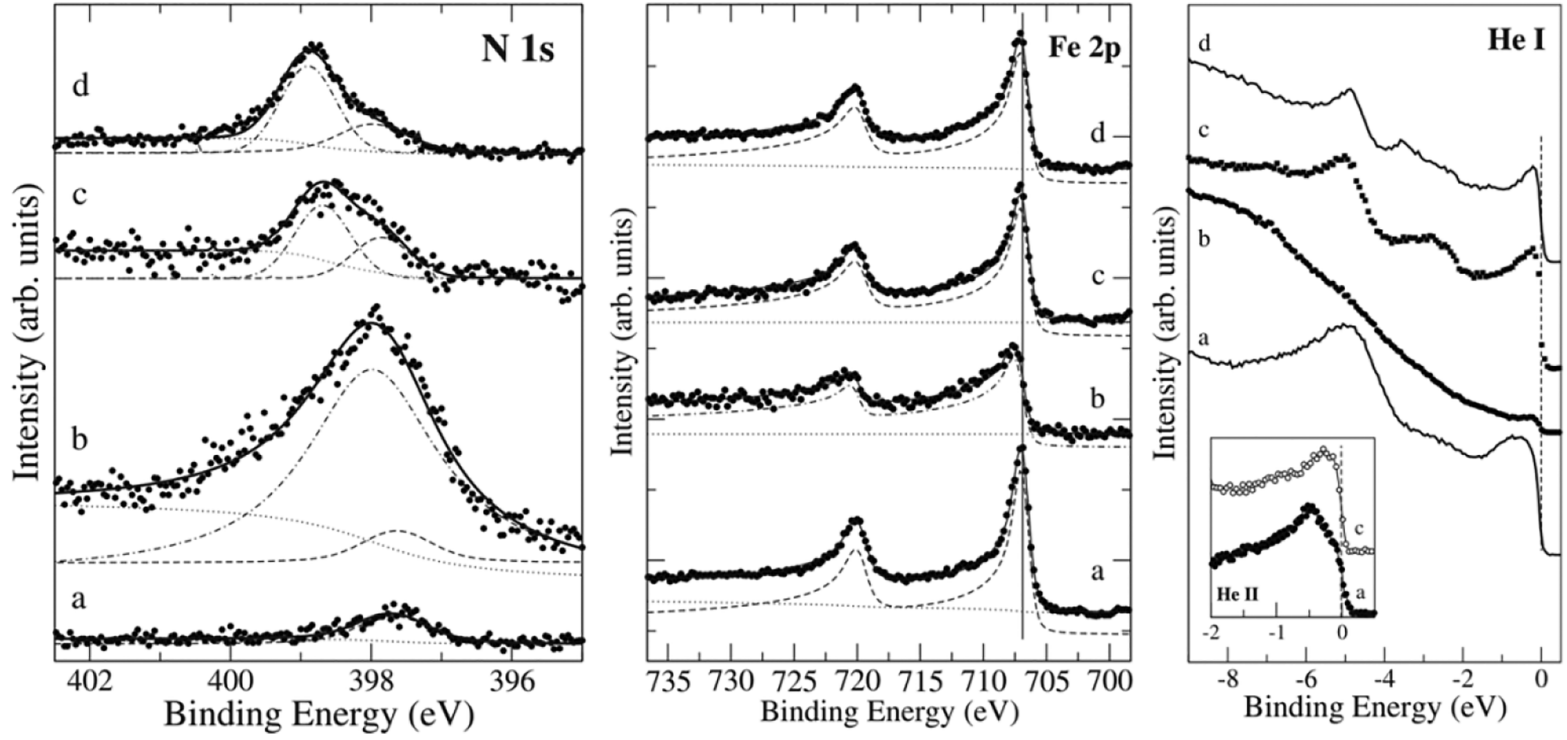
XPS core-level N 1s (left) and Fe 2p (middle) spectra, as well as UPS He I (right) and He II (inset) spectra of (a) nitrogen chemisorbed on Fe(001), (b) γ′′-FeN grown at 300 K on Cu(001), (c) the same iron nitride film as in (b) after annealing at 715 K, (d) γ′-Fe_4_N film grown on Cu(001). Reprinted with permission from ref (100). Copyright 2008 the American Physical Society.

Compared to the vast amount of theoretical data on the electronic properties of iron nitrides, there are very few experimental reports on this topic. The main methods used in these studies are XPS and UPS—the techniques which provide information on the core–electron levels and the valence band, respectively. Figure 8 presents exemplary N 1s and Fe 2p XPS spectra, as well as UPS spectra of N(2 × 2)/Fe(100) (chemisorbed), γ′-Fe_4_N/Cu(001), and γ′′-FeN/Cu(001)—the latter two representing a nitrogen-poor and a nitrogen-rich iron nitride phase, respectively. As can be seen, the Fe 2p signal is similar for all three systems (spectra a, c, and d), while the position and intensity of the N 1s signal changes, with the appearance of an additional component at ∼399 eV, corresponding to N in iron nitride. The Cu signals do not change in the process of iron nitride formation, eliminating the possibility of nitrogen reacting with the substrate.^98^ Notably, the position of the N 1s peak of iron nitrides differs significantly from those of other metal nitrides (usually this peak is located at around 397 eV, e.g., at 397.1 eV in the case of TiN,^148^ at 396.1 and 397.4 eV for CrN,^149^ 397.1 eV in the case of AuN,^150^ and 397.0 eV for GaN^151^). For the room temperature-deposited γ′′-FeN, the energy shift between the iron nitride component and the peak at 398 eV (corresponding to chemisorbed nitrogen) is small, but the intensity difference is large. What is more, the slight energy shift of the Fe 2p signals is also visible, suggesting a stronger interaction between the two elements. After annealing, the spectrum becomes similar to the one of γ′-Fe_4_N, indicating that the temperature of chemisorbed nitrogen desorption and iron nitride ordering was reached. Both phenomena—the N 1s shift to a lower binding energy and the Fe 2p shift to a higher binding energy—are attributed to the charge transfer between Fe and N. UPS He I (21.2 eV) spectra obtained for chemisorbed nitrogen and thin γ′-Fe_4_N film exhibit a prominent peak centered around −5 eV and corresponding to surface N 2p states. This feature is barely visible in the spectra obtained for room temperature-deposited γ′′-FeN due to a large background originating from photoelectrons inelastically scattered on a poorly-ordered surface.

Another technique that allows studying the local DOS is STS. This technique has superior spacial and energy resolution; however, it is highly focused on electronic states close to the Fermi level. STS studies were performed for ultrathin Fe_2_N and γ′-Fe_4_N films on Cu(001)^48,131^ and Cu(111).^105^ The DOS near the Fermi level was found to be characterized by two main electronic states: one at approximately −0.4 V, which is common for both substrates, and one at ∼0.4 V, which appears only for Cu(001). A comparison with the substrate spectra suggests that while the former might be attributed to the electron transfer from the substrate (i.e., the state also appears for pristine copper), the latter is related to iron nitride. Additionally, iron nitrides were found to exhibit an interface state at around 3–3.5 V, which is not present in the case of clean copper. Intensities and exact positions differed between the substrates, even though the morphology and composition of iron nitrides seem the same.

## 5 Magnetic Properties

**Figure 9:**
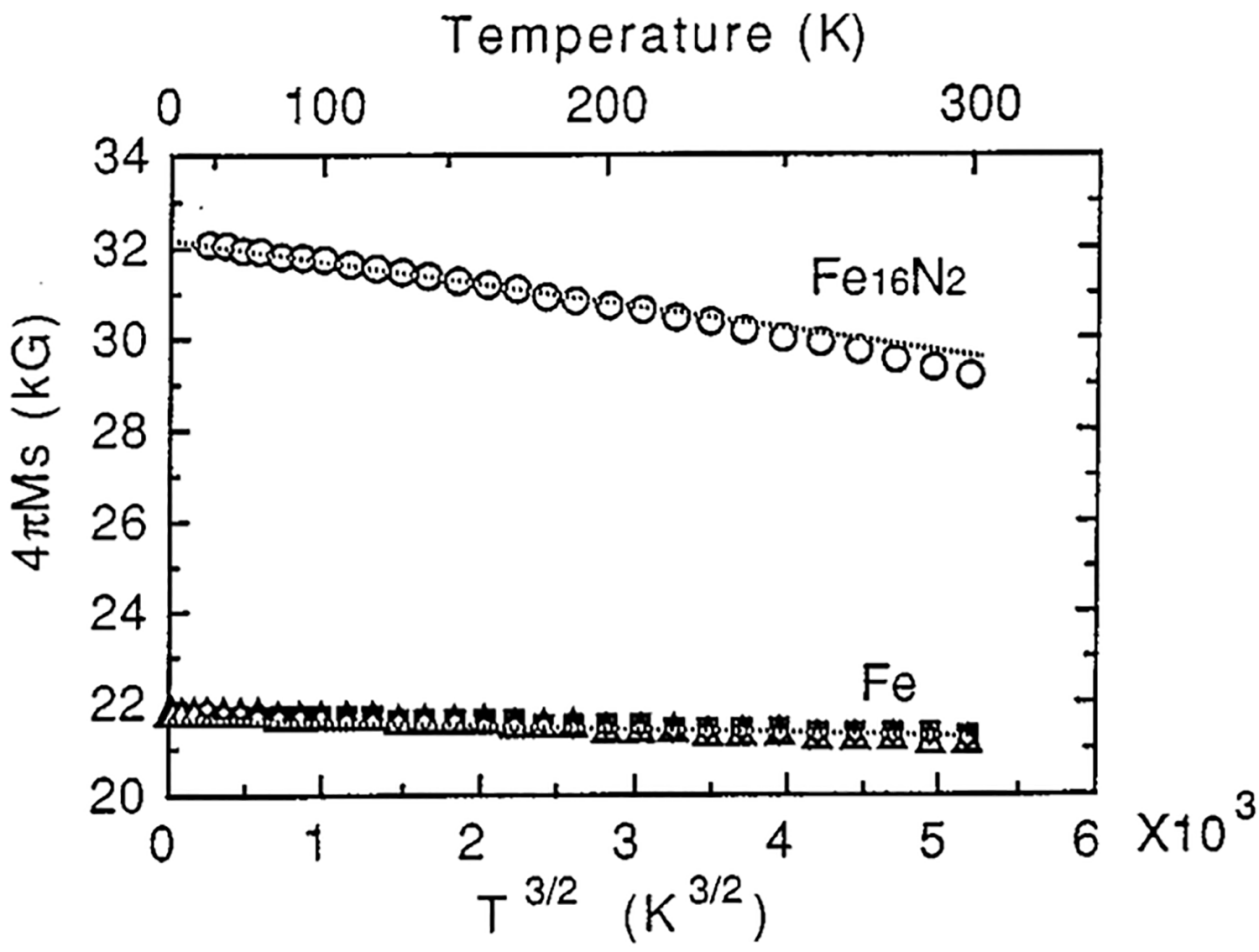
Temperature dependence of saturation magnetization of α′′-Fe_16_N_2_ and pure Fe films. Reprinted from ref (104) with permission. Copyright 1996 AIP Publishing.

Most iron nitrides, such as γ′-Fe_4_N, α′-Fe_8_N, or α′′-Fe_16_N_2_, are ferromagnetic and characterized by high Curie temperatures of >700 K. One of the phases, the γ′′′-FeN, is antiferromagnetic with a Néel temperature of 100 K. The basic information on magnetic orderings and order–disorder transition temperatures of the most common iron nitride phases were presented in Table 1 in section 1. The α′′-Fe_16_N_2_ attracts most of the attention thanks to its gigantic magnetic moment exceeding 2.9 μB/Fe atom (compared to 2.35 μB/atom of bulk bcc iron).^9,10,89,123^ This is far above the Slater–Pauling limit of 2.45 μB/atom, making it the largest magnetic moment per atom in binary rare-earth free compound. The biggest problem in the research and application of this material is the preparation of a pure α′′ phase which is prone to transform into a stoichiometrically similar, but much poorer in terms of magnetic saturation, α′-Fe_8_N phase. As for today, it is possible to obtain thin films of this compound with magnetic moment values reaching 3.5 μB/Fe atom at room temperature,^104^ which corresponds to a saturation magnetization of around 3 T (Figure 9; the result obtained for 34 nm-thick iron nitride film grown on GaAs(001)). The Curie temperature of this phase is estimated to be around 540 °C^10^ (based on the temperature dependence of the saturation magnetic field and approximation of the Langevin function). Unfortunately, this phase cannot be annealed to such a high temperature, as it decomposes above 400 °C (the change of saturation magnetization becomes irreversible).

**Figure 10:**
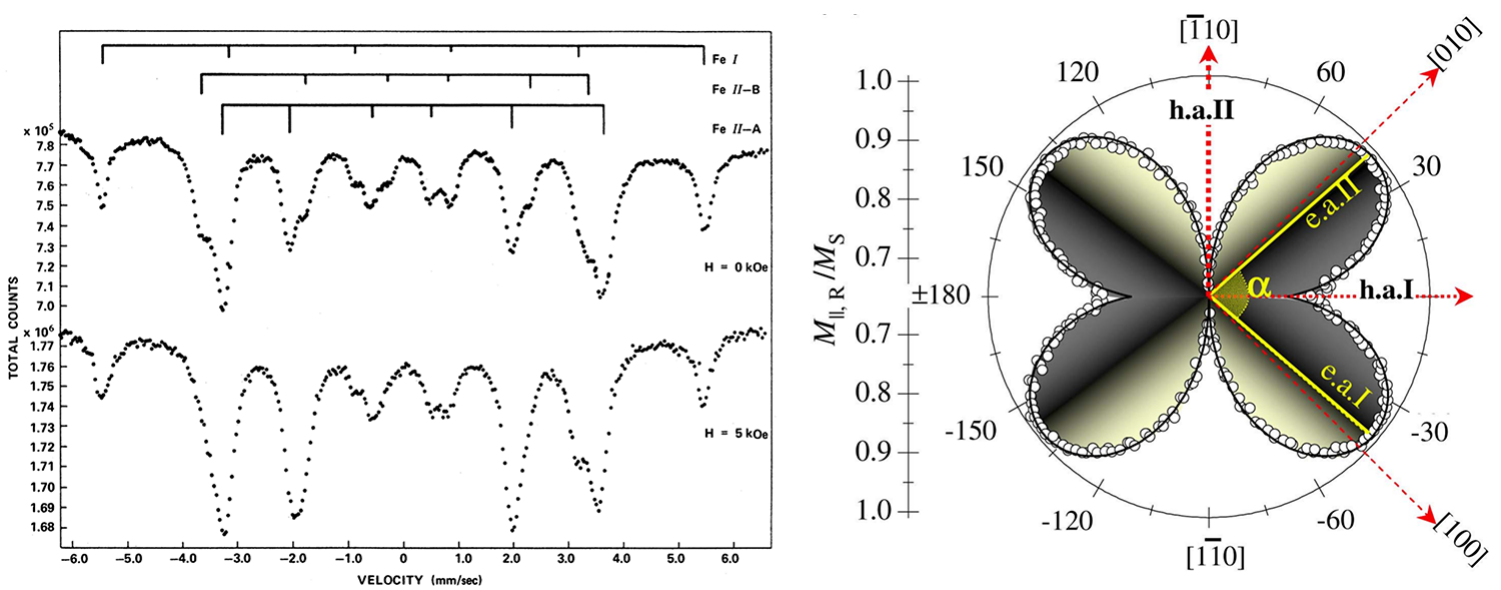
(Left) Mössbauer spectra recorded for a thin γ′-Fe_4_N film without (top curve) and with a 5 kOe magnetic field. Splitting of Fe-II atoms into Fe II-A and Fe II-B is visible in zero field but vanishes with an applied field. (Right) Polar *M*_R_ plot recorded for γ′-Fe_4_N film using Kerr magnetometry. Reprinted figures with permission from ref (153) (left) and (125) (right). Copyright 1971 (left) and 2008 (right) by the American Physical Society.

More interest, thanks to its stability and relative simplicity of growth, is devoted to γ′-Fe_4_N. This iron nitride phase exhibits a smaller (as compared to the α′′-Fe_16_N_2_) magnetic moment value of 2.0–2.2 μ_B_/Fe atom^62,138^—approximately 3.0 μ_B_ for Fe I atoms and 2.0 for Fe II (see Figure 2), being ferromagnetic with a Curie temperature of 767 K and a saturation magnetization of ∼1.8 T.^32,152^ Mössbauer spectroscopy studies revealed an additional split of the magnetic structure to signals originating from Fe II-A and Fe II–B atoms, with an intensity ratio of 2:1 and antiparallel orientation (Figure 10 (left)).^153^ This splitting disappears when a magnetic field is applied.

The orientation of easy axes and hard axes of the γ′-Fe_4_N film grown on Cu(100) was determined using Kerr magnetometry.^99,125^ Hard axes were found to be perfectly aligned with the in-plane crystallographic directions, i.e. along the [110] and [−110] crystal directions. The angles at which the easy axes occur depend on the anisotropy constants *K*_1_/*K*_2_ ratio within the film:^125^
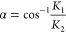


In the case of γ′-Fe_4_N, the easy axes are not orthogonal—the spread between them is approximately 81°, with the bisector of the angle pointing toward the [110] crystal direction (that is, the anisotropy constant *K*_1_ ≠ 0).^99,125^ Therefore, the angular dependence of remanence forms a butterfly-like structure (Figure 10 (right)). In contrast to α′′-Fe_16_N_2_, the γ′-Fe_4_N is characterized by a lower magnetic saturation, which can, however, be elevated by growing Fe/Fe_4_N multilayered structures.^11,128,154^ Such multilayers exhibit saturation magnetization, which is still lower than that of superior α′′-Fe_16_N_2_ but exceeds (by 32%) that of α-Fe and the Slater–Pauling limit.

The next iron nitride phase with higher nitrogen content, ε-Fe_*x*_N, also exhibits ferromagnetic ordering. Its Curie temperature strongly depends on the *x* parameter (nitrogen content) and falls between 525 K for Fe_3_N and 9 K for Fe_2_N.^29,155^ In the thin film form, the ε phase exhibits a much lower saturation magnetization than the nitrogen poorer iron nitride phases.^126^

The only orthorhombic iron nitride phase—ζ-Fe_2_N—is a weak ferromagnet with the Curie temperature strongly dependent on the dimensionality of the compound (bulk/layer/powder, etc.). In a thin film form, it varies from 35^61^ to 60 K,^156^ which is much higher compared to the bulk (4 K^60^). Even in a ferromagnetic state, it exhibits a much lower magnetic moment value per Fe atom (0.7 μB) compared to other iron nitrides. Similarly to the γ′-Fe_4_N phase, its magnetic characteristic can be improved through the formation of multilayers with pure iron.^120^

The high nitrogen content phases, γ′′- and γ′′′-FeN, differ significantly in terms of magnetic properties, as compared to other iron nitrides. CEMS studies revealed that zinc blende phase exhibits a single peak—indicative of paramagnetic ordering.^75^ The rock salt phase shows a sextet—which is characteristic of ferromagnetic and antiferromagnetic materials. In fact, the phase was confirmed to be antiferromagnetic, with a Néel temperature of 220 K.^127^ One of the first reported spectra obtained for iron nitride with approximately 50% nitrogen content indicated the coexistence of both phases;^102^ however, this interpretation was not definite. The zinc blende phase starts to show magnetic ordering only after nitrogen is partially desorbed and the nitride undergoes a phase transition into one of the phases with a higher iron content.^34^

## 6 Ultrathin Iron Nitride Films

Ultrathin films, i.e., films the thickness of which ranges from one to few crystal unit cells, are known to exhibit unique physicochemical properties originating from their low-dimensionality and the interaction with the underlying support. In general, the thinner the film, the stronger the influence of the substrate on its structure and properties (even though in some cases the film-substrate interaction may extend up to several nanometers into the film). The structure and properties of ultrathin films may be affected by epitaxial strain resulting from the lattice mismatch with the substrate, chemical interactions between the film and the support, the electronic interactions and—in the case of magnetic materials—magnetic interactions. Among the substrates most commonly used for ultrathin iron nitride films growth are MgO(001),^94^ Cu(001),^96^ Fe(001)^101^ (which is particularly interesting from the point of view of studies on nitrogen adsorption and surface nitride formation)^98^ and w-GaN.^122^

**Figure 11:**
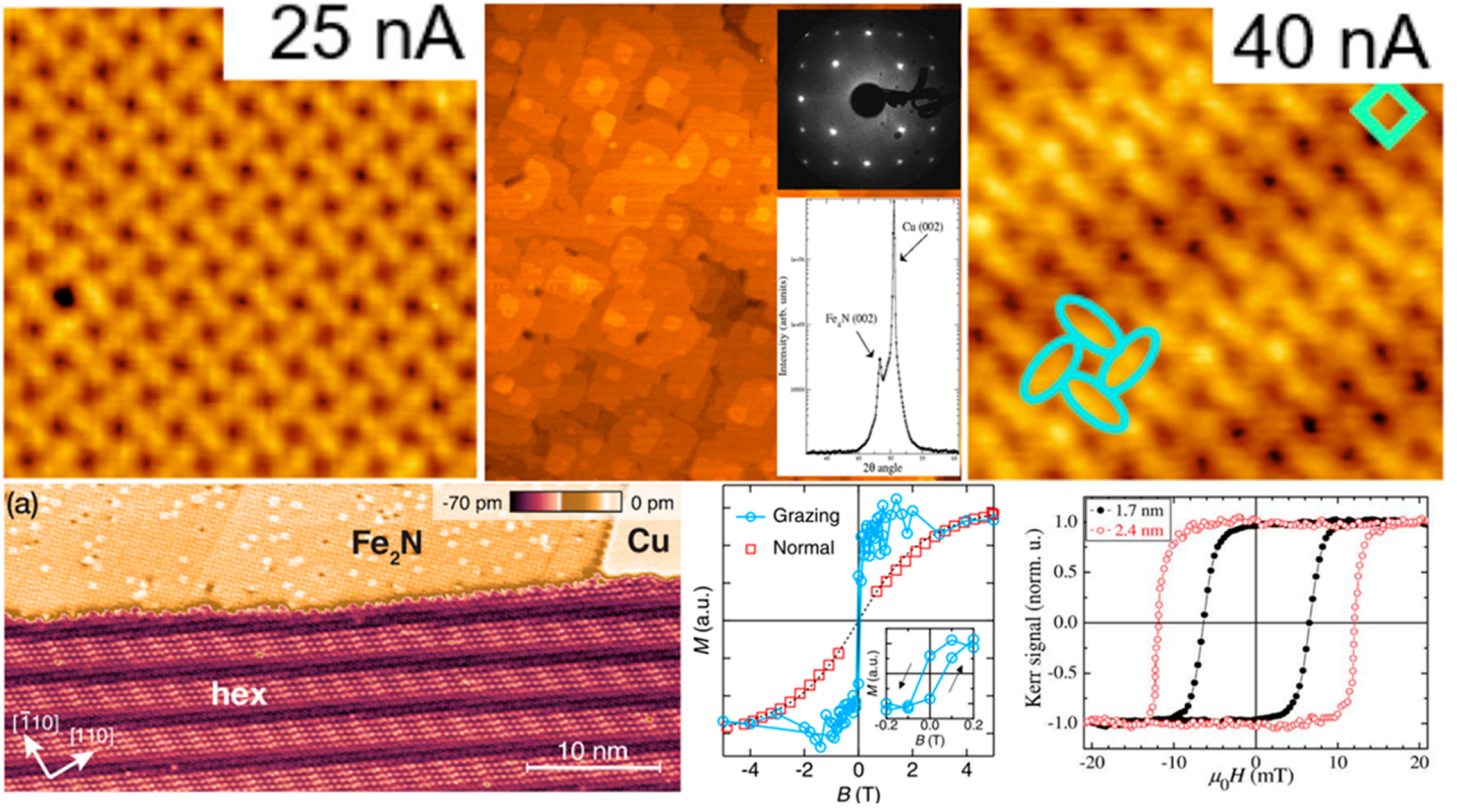
Top row: (left) Atomically resolved STM image (*V*_s_ = +50 mV, *I*_t_ = 25 nA) of Fe_2_N/Cu(001) with a *p4gm*(2 × 2) reconstruction; (middle) an image (*V*_s_ = +0.35 V, *I*_t_ = 470 pA) obtained for a thicker (18 Å) film with γ′-Fe_4_N stoichiometry (the corresponding LEED (110 eV) and XRD patterns are shown in the inset); (right) an atomically resolved STM image (*V*_s_ = +50 mV, *I*_t_ = 40 nA) of Fe_2_N/Cu(111). Bottom row: (left) A large-scale STM image (*V*_s_ = −0.1 V, *I*_t_ = 500 pA) showing the hexagonal reconstruction of FeN/Cu(001); (middle and right) magnetic hysteresis loops recorded using XAS and MOKE, respectively, for monolayer Fe_2_N (middle) and multilayer γ′-Fe_4_N (right) films. The (top-left, middle, right) and (bottom-left, middle) are reprinted figures with permission from ref (76) (top-left, right), (98) (top-middle), (130) (bottom-left), and (131) (bottom-middle). Copyright 2018 (top-left, right), 2007 (top-middle), 2017 (bottom-left, middle) by the American Physical Society. The (bottom-right) image is reprinted from ref (121) with the permission. Copyright 2009 AIP Publishing.

MgO(001) and Cu(001), due to their surface structure, are particularly suitable for growing thin γ′-Fe_4_N films.^94^ On dielectric MgO, the nitride adopts the cubic fcc structure of the substrate, which is the result of an only 10% lattice mismatch between the two materials (*a*_MgO(001)_ = 4.21 Å vs *a*_γ′-Fe4N(001)_ = 3.795 Å). Relaxation of the crystal lattice is observed in the [001] direction, leading to a *c*/*a* ratio of 1.01. However, the crystallographic directions [001] and [011] of the nitride and the substrate are not perfectly aligned, leading to the appearance of a small (<1°) tilt.^95^ The magnetic properties of the films are rather weakly influenced by the substrate, being ferromagnetic even at room temperature. In the case of Cu(001), the first layer usually represents half of the unit cell of γ′-Fe_4_N and therefore, it is often denoted as “Fe_2_N”.^48,157^ Ultrathin Fe_2_N and γ′-Fe_4_N films on Cu(001) may be either characterized by a *p4gm*(2 × 2) reconstruction—visible in STM images (Figure 11a) and LEED patterns^124^—or by a c(2 × 2) structure.^32^ Both structures are rotated with respect to the Cu(001) support by 45°, taking advantage of the small lattice mismatch between the surface lattice constant of γ′-Fe_4_N and the √2 interatomic distances in Cu(001) (*a*_Cu(001)_ × √2 = 3.606 Å). As revealed by AES, the structural difference between these two reconstructions lies in the termination of the iron nitride layer, where the *c*(2 × 2) structure exhibits a much stronger Fe signal in the spectra—pointing toward the iron-terminated layer, while the *p4gm*(2 × 2) films show stronger N peak—indicating a nitrogen-terminated structure.^32^ However, it has to be noted that the c(2 × 2) pattern appears also for atomic nitrogen chemisorbed on the iron surface,^98,100,101,121^ and therefore, the formation of an iron nitride compound, instead of an adsorbate layer, always has to be confirmed with different methods. The nucleation of γ′-Fe_4_N on Cu(001) proceeds in the form of islands which in STM appear to be embedded in the substrate,^97^ while subsequent layers grow in the form of terraces with square shapes (Figure 11b).^98^ If the substrate is rich in narrow terraces, it is possible to form, using the same preparation procedure, an iron nitride phase with hexagonal reconstruction.^130,134^ This reconstruction appears as a series of bright and dark stripes in STM images (Figure 11c). The regions are characterized by slightly different lattice constants, which is the result of the relaxation of strain originating from the lattice mismatch between the hexagonal iron nitride and the cubic substrate. While several iron nitride phases exhibit a hexagonal surface structure, in-depth geometrical and crystallographic analysis, presented in ref (134), points in favor of the γ′′-FeN phase. When annealed in UHV, the structure slowly undergoes phase transition, transforming into γ′-Fe_4_N. The temperature at which the surface is almost completely covered with the latter phase (as checked with LEED and XRD) is approx. 700 K.^100^ Interestingly, when Cu(111) is used as a substrate, the crystallographic orientation of the growing γ′-Fe_4_N films is the same as in the case of Cu(001), i.e., [001]. The combination of a hexagonal substrate and square film results in the formation of a Moiré superstructure, which is visible on STM images. The structure has the form of parallel darker and brighter stripes separated by approximately 1.7 nm with the atomic periodicity characteristic of γ′-Fe_4_N(001) (Figure 11d).^76,105^ The formation of γ′′-FeN on Cu(111) was not reported.

The electronic and magnetic properties of ultrathin γ′-Fe_4_N(001) films on Cu(001) exhibit a strong thickness dependence.^121,131^Figure 11e and Figure 11f show magnetic hysteresis loops obtained for films with monolayer and multilayer thickness, respectively. In all the cases, the films are characterized by an in-plane magnetic anisotropy; however, the coercivity values strongly depend on the film thickness. Notably, in contrast to iron nitride films grown on Si and SiO_2_ substrates, described in section 3, the films on Cu(001) and MgO(001) do not undergo a phase transition with increasing thickness.

Fe(001) is another interesting substrate for the growth of ultrathin iron nitride films, as it does not promote to the formation of the γ′-Fe_4_N iron nitride phase but the zinc blende γ′′-FeN structure.^101^ The growth of well-ordered films may be obtained by exposing the substrate to atomic nitrogen even at room temperature. This reveals that N atoms can easily diffuse into Fe(001), forcing the adsorption-induced reconstruction of the near-surface iron layers. The films grow rotated by 45° with respect to the substrate, as in this direction ZB-FeN encounters a relatively small lattice mismatch (6%, as *a*_ZB-FeN_ = 4.307 Å and *a*_Fe(001)_ × √2 = 4.053 Å). The structure is not very thermally stable, as it decomposed upon annealing at 680 K. The films were found to be paramagnetic, as expected for this iron nitride phase.

On w-GaN(0001), both single crystal^122^ and thin films grown on sapphire,^158^ the epitaxial growth of FeN is possible thanks to the nearly matching distances of Fe–Fe in zinc blende FeN (3.041 Å) and Ga–Ga in w-GaN (3.189 Å). Iron nitride grows following the Stranski–Krastanov (SK) mode, with the determined critical thicknesses of 2 nm in the case of a single-crystal w-GaN substrate and 0.5 nm for GaN/sapphire. The reason for such a difference may lie in the different growth method used—sputtering for GaN(0001) and MBE for GaN/sapphire. On the other hand, both studies revealed the out-of-plane lattice spacing evolution during the film growth, with the *d* spacing varying from 2.7 to 2.9 Å at the early stages and stabilizing at ∼2.65 Å for thicker films. Such an evolution may be the result of epitaxial strain relaxation in the thin film. Both articles report that a single-phase γ′′-FeN film was obtained; however, the article by Lin reports that at the beginning of iron nitride growth (∼0.5 ML), the RHEED pattern behavior suggests a transition from the w-FeN phase towards the fcc one (either rock salt or zinc blende).^158^

## 7 Potential Applications

**Figure 12:**
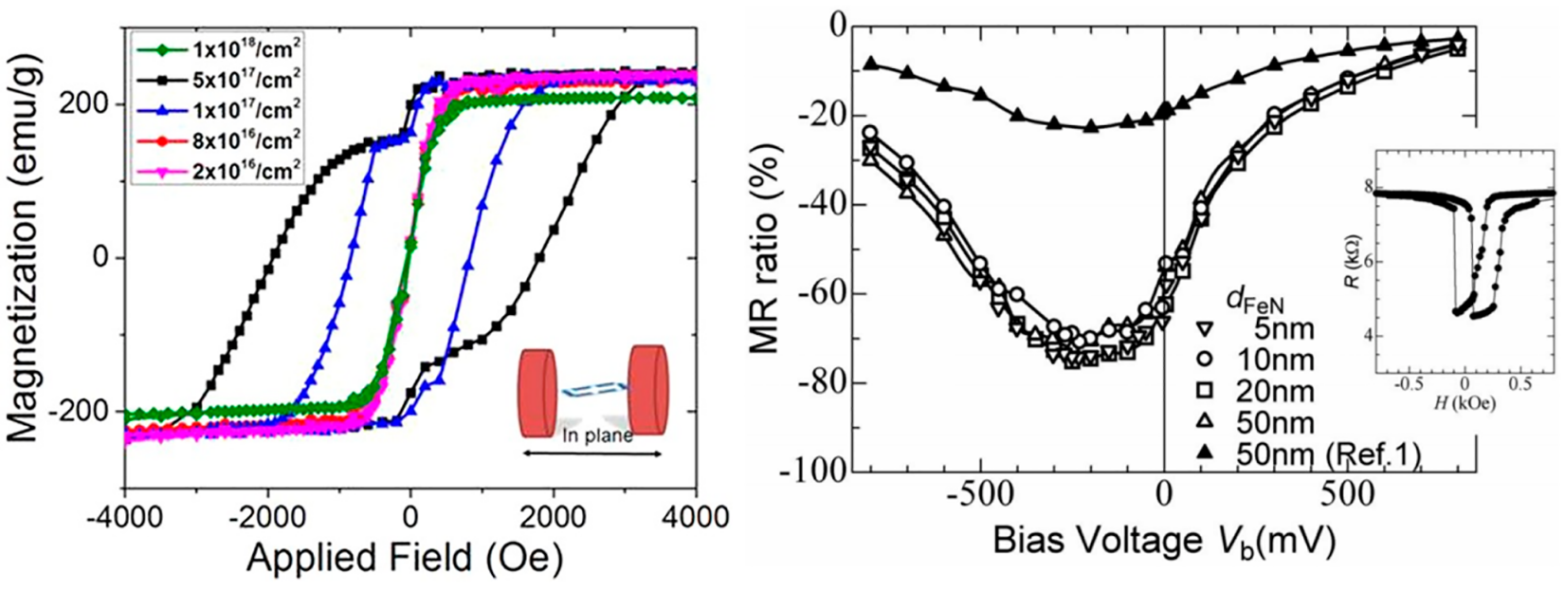
(Left) In-plane magnetic hysteresis loops obtained for iron foils subjected to different nitrogen ion implantation fluences. (Right) Bias-voltage dependence of the tunneling magnetoresistance ratio for Fe_4_N/MgO/CoFeB magnetic tunnel junctions with varying γ′-Fe_4_N layer thickness. The left image is reprinted with permission from ref (129) under the terms of the Creative Commons CC BY license. Published by Springer Nature 2016. The right image is reprinted from ref (78) with the permission. Copyright 2009 AIP Publishing.

The first and foremost potential application of iron nitrides is related to the α′′-Fe_16_N_2_ phase, which is the strongest known permanent magnet.^159^ Its high saturation magnetization and magnetocrystalline anisotropy make it a potential candidate for substituting rare-earth elements containing alloys in electrotechnical devices. One requirement for such an application is, however, to stabilize this iron nitride phase in the form of a bulk material instead of a thin film. In fact, it has been reported that nitriding an iron foil through nitrogen ion implantation results in its conversion into α′′-Fe_16_N_2_, as manifested by hard magnetic properties.^129^ These properties were found to change based on ion implementation fluence, as revealed by the recorded hysteresis loops (Figure 12 (left)).

Thin iron nitride films are also promising materials for application in semiconducting and spintronic devices. Multilayer CoFeB/MgO/Fe_4_N/Cu(001) structures were shown to exhibit up to −75% inverse tunnel magnetoresistance at room temperature (Figure 12 (right)),^78^ which is a good value for potential application in magnetic logic circuits. Additionally, thermal decomposition of FeN (nonmagnetic metallic) and Cu_3_N (semiconducting^160^) can be used for precise, laser-based lithography of spin valves.^121^ More precisely, FeN/Cu_3_N/Fe_4_N/Cu(001) (nonmagnetic/semiconductor/magnetic/substrate) was proposed as a base for local laser irradiation which increases the temperature and promotes nitrogen diffusion, changing the local structure into Fe_4_N/Cu/Fe_4_N one (magnetic/nonmagnetic/magnetic/substrate).

Technological application of iron nitrides does not necessarily have to utilize their magnetic properties. Transition metal nitrides—including iron nitrides—are also investigated as potential electrodes in lithium ion batteries, allowing for higher capacities and charging speeds. As an example, nanocrystalline thin γ′′-FeN film sputter-deposited onto a Cu foil was found to exhibit a reversible electrochemical reduction of iron nitride in the presence of lithium into pure iron and lithium nitride. The performance of the system relying on this reaction was found to be superior to most transition metal nitrides, with a high specific capacity and structural stability after charge/discharge cycling.^133^

Another field of potential applications is heterogeneous catalysis. There are several works focused on catalysts consisting of iron nitride-covered nanoparticles and powders which are active in various chemical reactions. The mechanisms of such reactions could be determined through model studies carried out on thin-film systems, which—to the best of our knowledge—have not been performed so far. An example of an iron nitride-catalyzed chemical process is the so-called oxygen reduction reaction (ORR)—a reaction in which an O_2_ molecule is converted to H_2_O or H_2_O_2_. This process is utilized, e.g., in fuel cells,^161^ with costly platinum-based catalysts used in common designs.^162^ In order to decrease the cost of ORR catalysts, several materials consisting of Earth-abundant elements were tested, including compounds of iron nitride and nitrogen-doped graphene (NG).^163−169^ As it appears, FeN/NG promotes the ORR reaction with the effectiveness comparable^167,169^ or even superior^168^ to that of platinum-based catalysts. In the case of this catalyst, oxygen molecules are adsorbed onto iron nitride nanoparticles and the O–O bonds are weakened due to the interaction of oxygen with iron. Another reaction in which iron nitrides are used as a catalysts is the decomposition of hydrazine (N_2_H_4_). This process is utilized in spacecrafts—in thrusters responsible for controlling the orbit and altitude. Current state-of-the-art catalyst of this reaction is composed of iridium supported on aluminum oxide, and hence, there are significant efforts to substitute this rare and expensive noble metal with a more abundant material. The studies revealed that ε-Fe_3_N constitutes a superior alternative catalyst with a conversion rate reaching 100%.^170,171^ Zheng et al. suggest that the lattice expansion in iron nitride (as compared to pristine iron) influences the electronic structure of the compound, resulting in the DOS at the Fermi level comparable to that of noble metals–thus explaining the high catalytic activity.^170^ Interestingly, the same catalyst can be used for further decomposition of ammonia—one of the products of this reaction. At a sufficiently high temperature (>500 °C), ammonia decomposes to hydrogen and nitrogen, with a conversion rate reaching also 100%. Recently, it has also been discovered that iron nitrides promote the synthesis of higher alcohols (ethanol and heavier) from syngas (mixture of CO and H_2_).^172^ Three different Fe_*x*_N samples (*x* = 2, 3, 4) were obtained by nitriding α-Fe. Under high-temperature and high-pressure conditions, and a prolonged reaction time (210 °C, 2 MPa, 2000 h), all the samples exhibited an ∼30% CO conversion rate. Among the products, up to 30% were different alcohols, with 50% of them being higher alcohols. This selectivity is superior compared to currently used industrial catalysts.

## 8 Summary and Outlook

Iron nitrides are fascinating materials characterized by unique magnetic properties similar to that of permanent rare-earth magnets. The number of possible crystalline Fe–N phases, differing by the nitrogen content, is overwhelming, with some of the theoretically predicted ones still waiting to be experimentally synthesized. Even though the materials are the subject of fundamental and applied research since the beginning of the 20th century, the relations between the structure and properties of different iron nitride phases are still not well understood. This includes not only the origin of their magnetic properties, but also superior mechanical properties, electronic properties, and catalytic activity, comparable to that of noble metals. Once this knowledge is gained, the materials can find applications in many technological fields, ranging from spintronics, through protective coatings, to catalysis and energy conversion.

As far as thin iron nitride films are concerned, most of the known crystalline phases can be stabilized in a thin-film form; however, some require a specific substrate and/or preparation method. This constitutes a significant drawback from the point of view of potential applications. In most reported cases, thin films representing the γ′-Fe_4_N phase, and growing in the [001] crystallographic direction, were obtained. Less commonly, the α′′-Fe_16_N_2_, γ′′-FeN, and ε-Fe_3-*x*_N structures were synthesized. The formation of other phases, such as α′-Fe_8_N, γ′′′-FeN, or ζ-Fe_2_N, was rarely observed. On more “technical” SiO_2_/Si substrates or metal foils, the films were found to grow in a multiphase and polycrystalline form. Therefore, the ways to synthesize single-crystal and single-phase films of different iron nitrides, including the “exotic” ones, such as ferromagnetic α′-Fe_8_N and ζ-Fe_2_N phases or the antiferromagnetic γ′′′-FeN phase, and on cost-effective substrates, have to be developed. When it comes to the growth techniques, both PVD (PLD, magnetron sputtering, MBE) and CVD (including ALD) methods were successfully used. In the case of PVD, single-phase and single-crystal films are obtained only on single-crystal substrates (e.g., MgO(001) or Cu(001)). To obtain such films, Fe deposition in the presence of nitrogen plasma and hydrogen (that allows binding the excess of nitrogen) seems to be the most effective way. For ultrathin films, sputtering the substrate with N^+^ ions, depositing Fe, and annealing in UHV can be also used. Such films show thickness-dependent electronic and magnetic properties, which is interesting from the point of view of potential applications.

Looking into the future and taking into account the fact that iron nitrides are electrically conducting and exhibit interesting magnetic properties—either ferro- or antiferromagnetic—the most promising field of application seems to be spintronics, with iron nitrides as building blocks of spin-manipulating devices. In this context, the easily obtainable γ′-Fe_4_N phase, as well as the α′′-Fe_16_N_2_ phase, with its superior value of magnetic moment per Fe atom of 2.9 μB, attract particular attention. It is also worth noting that iron nitrides are composed of Earth-abundant elements, ensuring—with proper technological facilities—low fabrication costs of potential devices. However, the application of these materials will not be immediate since they are almost nonexistent in nature, which is the result of their inferior stability compared to, for example, iron oxides. Therefore, it is crucial to discover ways to stabilize these compounds and ensure their structural integrity before, during, and after industrial processing, which is one of the main challenges in the field. Another challenge, strictly connected to this, is developing an effective way (preferably CVD-based) for large-scale synthesis of single-phase and single-crystal thin iron nitride films. In conclusion, even though the properties of iron nitrides seem marvelous, there is still much to be done—both with respect to fundamental and applied studies—in order to understand their physicochemical characteristics and apply them in real-life commercial devices. The works are ongoing.

## List of Acronyms Used in Table 2

AES: Auger electron spectroscopy
AFM: atomic force microscopy
CEMS: conversion electron Mössbauer spectroscopy
EDX: energy-dispersive X-ray spectroscopy
EELS: electron energy loss spectroscopy
EIS: electrochemical impedance spectroscopy
ERD: elastic recoil detection
FESEM: field emission scanning electron microscopy
FMR: ferromagnetic resonance
GIXRD: grazing incidence X-ray diffraction
HX-PES: hard X-ray photoelectron spectroscopy
LEED: low energy electron diffraction
LEIS: low energy ion scattering
MFM: magnetic force microscopy
ML: monolayer
MOKE: magneto-optical Kerr effect
MOMM: magneto-optic microscope magnetometry
NRS: nuclear resonant scattering
NRP: nuclear reaction profiling
PCAR: point contact Andreev reflection
PECVD: plasma enhanced chemical vapor deposition
PPMS: physical property measurement system
PNR: polarized neutron reflectivity
RBS: Rutherford backscattering spectrometry
RHEED: reflection high energy electron diffraction
RMS: root-mean-square
SAXS: small-angle X-ray scattering
SIMS/TOF-SIMS: (time-of-flight) secondary ion mass spectroscopy
SRPES: synchrotron radiation photoelectron spectroscopy
TEM/STEM: (scanning) transmission electron microscopy
STM: scanning tunneling microscopy
STS: scanning tunneling spectroscopy
SQUID: superconducting quantum interference device
UPS: ultraviolet photoelectron spectroscopy
UV–vis: ultraviolet–visible spectroscopy
VNA: vector network analyzer
VSM: vibrating sample magnetometry
XANES: X-ray absorption near-edge structure
XAS: X-ray absorption spectroscopy
XMCD: X-ray magnetic circular dichroism
XRR: X-ray reflectivity
